# Research on the sliding friction associated spur-face gear meshing efficiency based on the loaded tooth contact analysis

**DOI:** 10.1371/journal.pone.0198677

**Published:** 2018-06-26

**Authors:** Hao Dong, Zhi-Yu Liu, Ling-ling Duan, Ya-hui Hu

**Affiliations:** 1 School of Mechatronic Engineering, Xi’an Technological University, Xi’an, China; 2 Shaanxi Hualu Chemical Green Environmental Protection Co.Ltd., Xi’an, China; Lanzhou University of Technology, CHINA

## Abstract

In order to solve the problem of Meshing Efficiency of spur-face gear sliding friction, a method for calculating the Meshing Efficiency of Spur-Face gear is proposed based on Elastohydrodynamic lubrication (EHL) theory. Through the Tooth Contact Analysis (TCA) and Loaded Tooth Contact Analysis technique (LTCA) method, the meshing process of the Spur-Face gear was simulated. The calculation model of Sliding friction coefficient was established by using non Newtonian quasi steady thermal Elastohydrodynamic lubrication (TEHL) theory, and the calculation model of Meshing Efficiency of Spur-Face gear was established. The influence of input torque and rotational speed on Meshing Efficiency is analyzed. The results show that Sliding friction coefficient is an important factor affecting the Meshing Efficiency of gears. Sliding friction coefficient is not the same at different positions of the tooth surface. Sliding friction coefficient is affected by input speed and input torque. This method provides a theoretical basis for further optimization calculation of Spur-Face gear.

## Introduction

Gear drive as the most extensive mechanical transmission line, the transmission efficiency has important significance for saving energy. Generally speaking, the mechanical efficiency is usually determined by a specific test method, but in the gear transmission, the efficiency of the gear transmission is generally calculated using the Meshing Efficiency method. Gear transmission power loss mainly includes sliding friction power losses, rolling friction power loss and windage loss and Churning oil loss. The sliding friction power loss is the critical factor that affects the Meshing Efficiency of the gear, and the influence of rolling friction power loss is small, and the calculation ignores its influence. In the high speed gear transmission, windage loss and Churning oil loss are also critical factors affecting the gear Meshing Efficiency. In this paper, the effect of sliding friction power loss on engagement efficiency is only considered.

A lot of research on the Meshing Efficiency of the gears have been done at home and abroad. Kolivand and Kahraman [1] proposes a new spiral bevel and hypoid gear mechanical efficiency model for both face-milling and face-hobbing type cutting methods. The proposed efficiency model combines a computationally efficient contact model and a mixed EHL based surface traction model to predict friction power losses. Pedro Marques [2] developed an analytical model relying on the ISO 6336 maximum teeth stiffness and a parabolic single tooth stiffness per unit of single line length. The proposed model is of straight forward implementation, very little computational cost and yields promising results. Ankur Saxena[3] proposes a computer simulation based approach to study the effect of time varying friction coefficient on the total effective mesh stiffness for the spur gear pair. Xiaogang Zhang[4] considered the asperity interaction friction to result from either the boundary film friction or solid-to-solid ploughing and adhesion friction depending on the local contact and deformation conditions. Yuansheng Zhou [5] proposesed a new method to calculate gear speed ratio, velocity, torque and power based on hypergraph and matrix operation. Systematic efficiency computation is carried out by following the power flow, and power loss equations on each node are derived via an approach based on self-rotation relative power. Charles Nutakor [6] developed a composite power loss model combining a non-uniform load distribution model with a local friction coefficient at any point of contact and oil drag formulation. Diez-Ibarbia[7] analyzed the effect of the friction coefficient on the efficiency of spur gears with tip reliefs. Ziegltrum and Stahl [8]proposesed transient TEHL simulation model based on a finite element formulation and implemented in multiphysics software applied to study the TEHL contact along the path of contact of spur gears with a focus on load-dependent gear loss of different lubricants. Kahraman [9] proposesed an experiment to study investigates the contributions of the key components of load-independent (spin) power losses of planetary (epicyclic) gear sets. Pedro[10–12] introduced gear load sharing models for spur and helical gears taking into account elastic and frictional effects allowing to do more refined estimations of gear friction losses. A numeric power loss model simulating all the relevant power loss mechanisms was implemented, aiming to evaluate the relative impact of each power loss component. Franco Concli [13] developed a specific power loss calculation-tool that enables a good prediction accuracy with reasonable computational efforts, through considering the importance of such topic for gearbox manufacturers. Li Jianying [14] analyzed the power distributions of the complex and closed planetary gears transmission and calculated the overall transmission efficiencies under considering power losses or not. Sheng Li [15] proposesed a thermal tribo-dynamic mechanical power loss model for spur gear Pairs. Talbot and Kahraman [16] proposesed a methodology that implements a family of models to predict total power loss of planetary gear sets including primary mechanical and spin loss components with the assumption that these components of power losses are independent of each other. Xu H and Kahraman [17–19] proposed a computational model for the prediction of friction-related mechanical efficiency losses of parallel-axis gear pairs, and proposed a model to predict friction-related mechanical efficiency losses of hypoid gear pairs. The model includes a gear contact model, a friction prediction model, and a mechanical efficiency formulation. Wang [20] proposed a computational model to analyze the power losses of sliding friction based on meshing characteristic of double helical gears. Wang W Z [21] developed the EHL model of the involute helical gears by simplifing the contact between tooth surfaces in the mesh of a pair of helical gears into a finite line-contacts lubrication problem.

However, in the prior studies, the friction coefficient is mostly based on the average or by semi empirical formula. With the development of the TEHL theory and the corresponding multigrid algorithm, the numerical solution of EHL and calculation speed has been greatly improved. The authors use Tooth Contact Analysis and Loaded Tooth Contact Analysis to obtain the meshing paths and load distributions of the gears. The Sliding friction coefficient of the contact line at each point is get by using TEHL theory, and then the sliding friction power loss of the gear drive is obtained.

## Sliding friction power loss calculation

**Fig 1:**
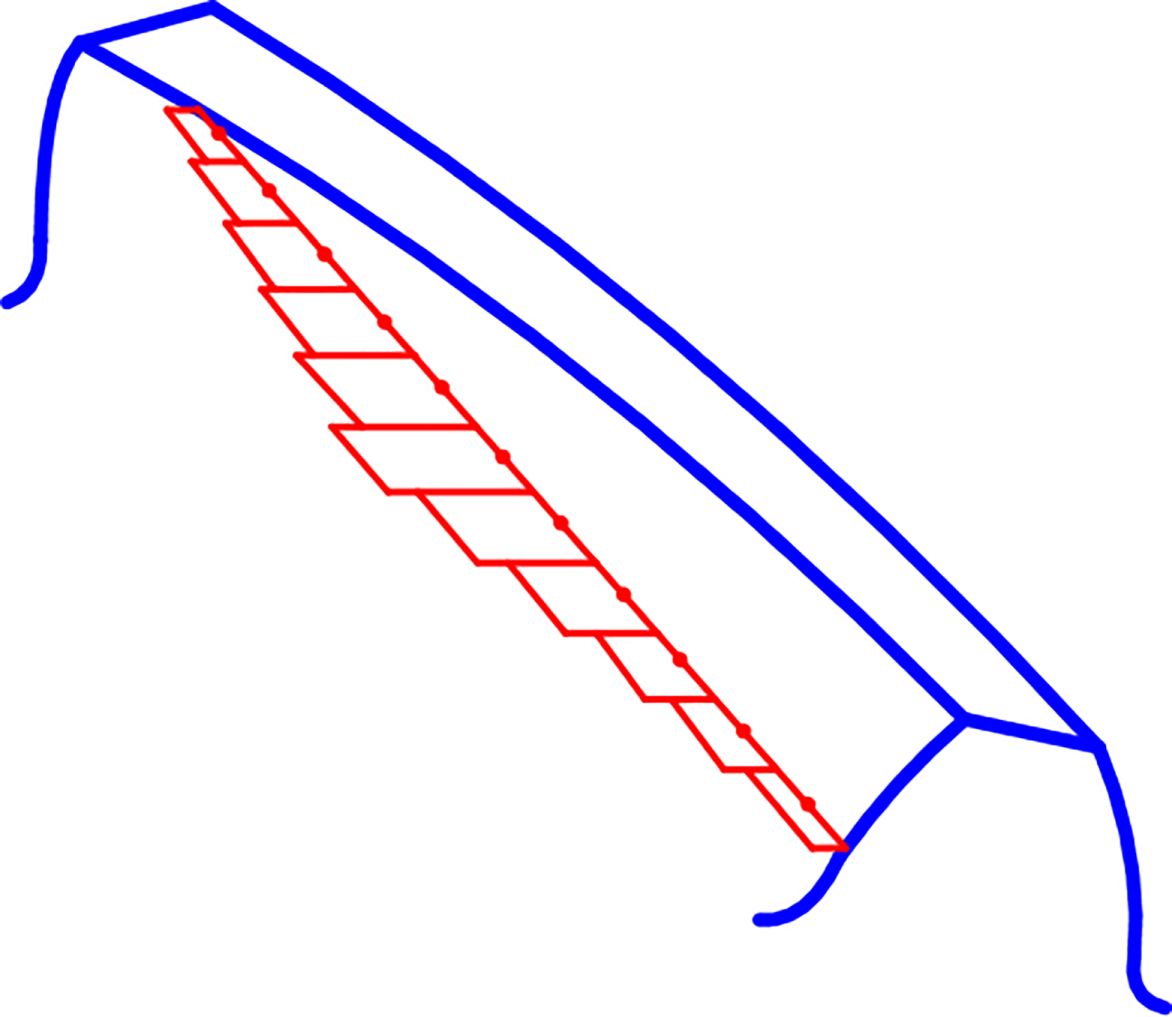
Schematic diagram of discrete contact line.

The parameters such as relative sliding speed and the normal load on the contact line are continuously changing, and the contact lines are dispersed into finite sections as showed in **Fig** 1. The parameters of each point on the meshing line are expressed by midpoint parameters of each segment. The sum of the sliding friction power loss from each discrete contact line approximately represents the sliding friction power loss of the instantaneous contact line.

**Fig 2:**
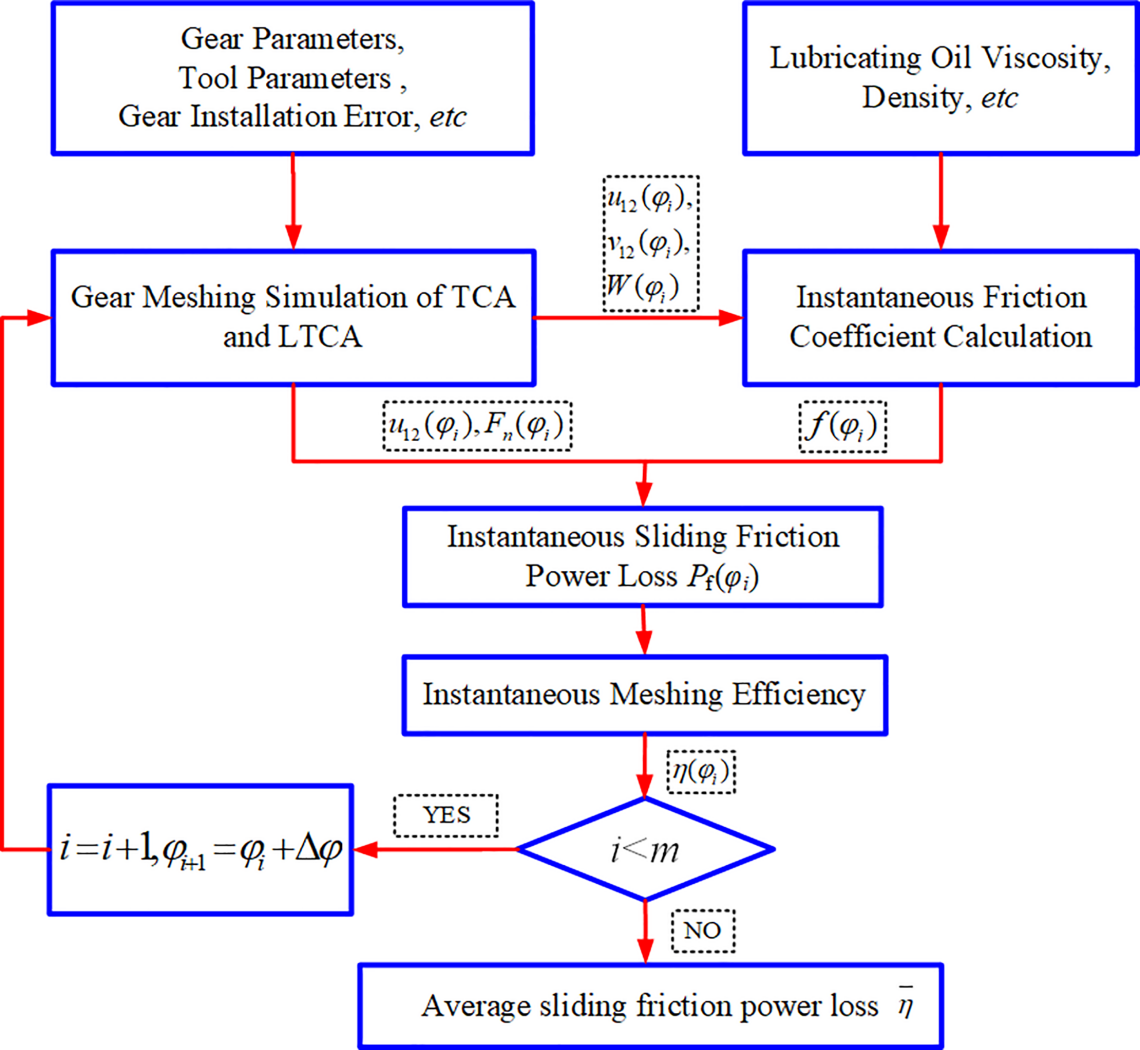
Calculation program of sliding friction power loss.

The sliding friction power loss calculation process of Spur-Face gear is illustrated in **Fig** 2. It is primarily composed of gear meshing simulation (TCA, LTCA), instantaneous friction factor calculation and sliding friction power loss calculation. The Tooth Contact Analysis (TCA) of Spur-Face gear is a technique for simulating meshing engagement [22]. The meshing process of spur-face gear is simulated by gear contact analysis, and the position vector of the midpoint of each discrete contact line can be obtained. The Loaded Tooth Contact Analysis (LTCA) of Spur-Face gear is a method of numerical simulation for the meshing process of gear teeth, which can obtain the normal load and load density at each discrete point in this paper [23].

Taking Δ*φ* as the step size, a meshing cycle is divided into *m*^th^ meshing positions, and the corresponding angles of the gears are *φ*_*i*_(*i* = 1,2,3,…,*m*) respectively. In the meshing position *φ*_*i*_, through the calculation of TCA and LTCA, we can get the normal load and load density at the discrete points at each meshing line at this position *φ*_*i*_. The sliding friction factor of the discrete points in the elastohydrodynamic lubrication state can be obtained by the equivalent radius of curvature, the relative sliding velocity, the normal load, the load density and the dynamic viscosity of the lubricating oil. The instantaneous sliding friction power loss at meshing position *φ*_*i*_ can be obtained from the normal load, relative sliding speed and sliding friction factor obtained above. By using the same method, the instantaneous sliding friction power loss of each meshing position in the meshing period is obtained, and then the average sliding friction power loss of the spur-face gear is obtained.

Through the calculation of TCA, we can get the instantaneous meshing point at any meshing position and the discrete points on the contact ellipse long axis of the gear.

**Fig 3:**
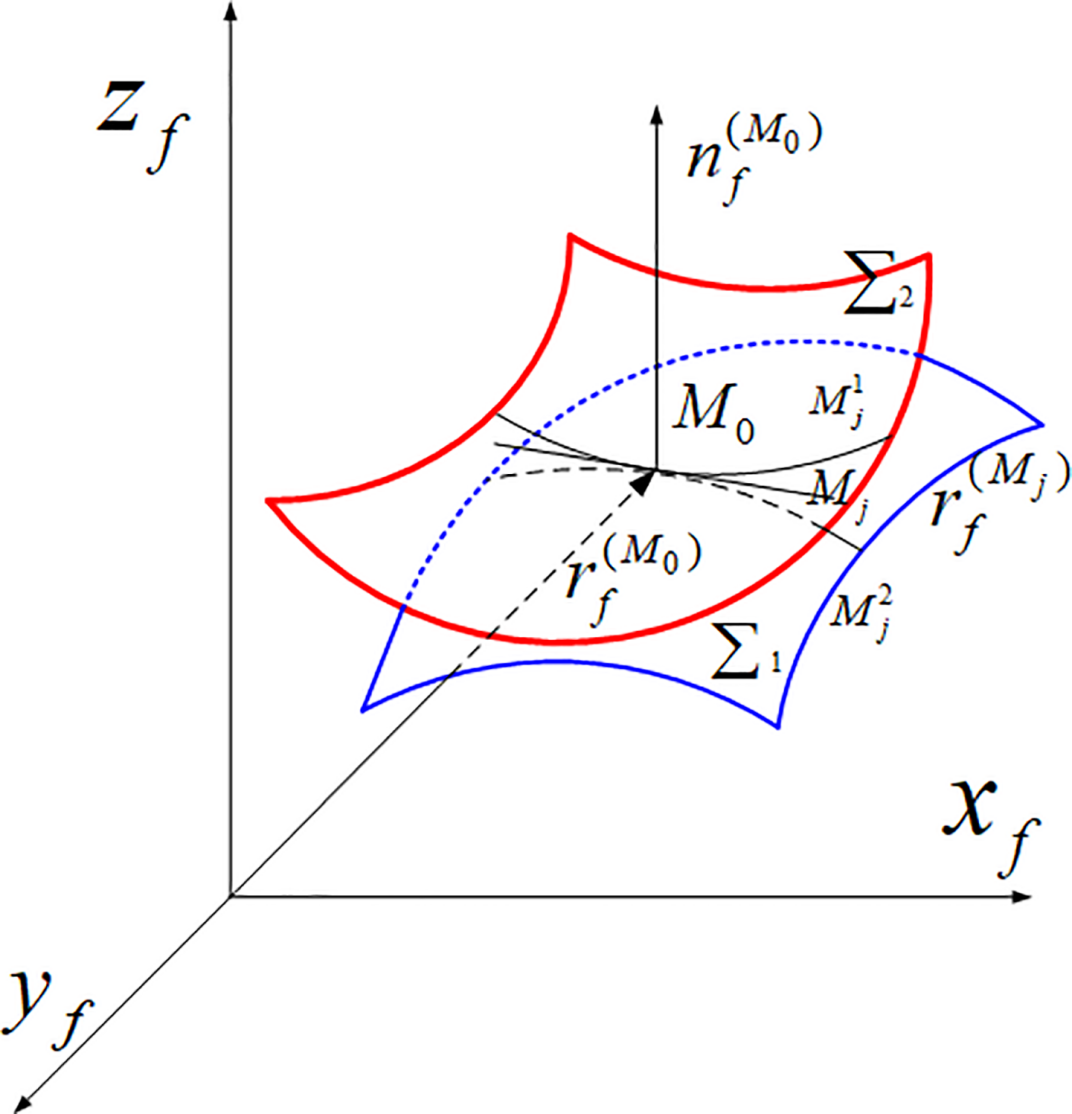
Discrete points on the long axis of contact ellipse.

As shown in **Fig** 3, in the fixed coordinate system *S*_*f*_, *∑*_1_ and *∑*_2_ represent the tooth surface of pinion and gear, *M*_0_ is instantaneous meshing point of the tooth surface *∑*_1_ and ∑_2_ at meshing position *φ*_*i*_, $r_{f}^{\left(M_{0}\right)}$ is the position vector of *M*_0_, $n_{f}^{\left(M_{0}\right)}$ is unit normal vector of *M*_0_, *M*_*j*_ is a discrete point for the long axis of the ellipse, $r_{f}^{\left(M_{j}\right)}$ is the position vector of *M*_*j*_, $r_{f}^{\left(M_{j}\right)}=r_{f}^{\left(M_{0}\right)}$ *+vector*(*M*_0_*M*_*j*_).

Draw a parallel line through point *M*_*j*_ and pass through the normal vector $n_{f}^{\left(M_{0}\right)}$ of the two tooth surface of *∑*_1_ and ∑_2_. The two intersection points $M_{j}^{1}\;(x_{j}^{1},y_{j}^{1},z_{j}^{1})$ and $M_{j}^{2}\;(x_{j}^{2},y_{j}^{2},z_{j}^{2})$ are respectively obtained. $M_{j}^{1}\;(x_{j}^{1},y_{j}^{1},z_{j}^{1})$, $M_{j}^{2}\;(x_{j}^{2},y_{j}^{2},z_{j}^{2})$ and $n_{f}^{\left(M_{0}\right)}$ conforms to the following formula:

$$\{\begin{array}{c} {}[y_{j}^{1}(u_{1},\theta_{1})-y_{j}]n_{z}-[z_{j}^{1}(u_{1},\theta_{1})-z_{j}]n_{y}=0\\ {}[x_{j}^{1}(u_{1},\theta_{1})-x_{j}]n_{z}-[z_{j}^{1}(u_{1},\theta_{1})-z_{j}]n_{x}=0 \end{array}$$ (1)

Here, *n*_*x*_, *n*_*y*_, *n*_*z*_ are respectively the projection of $n_{f}^{\left(M_{0}\right)}$ to each coordinate axes of the fixed coordinate system *S*_*f*_. *x*_*j*_, *y*_*j*_, *z*_*j*_ are respectively the projection of $r_{f}^{\left(M_{j}\right)}$ to each coordinate axes. *u*_1_ and *θ*_1_ are the tooth surface parameters. Through solving the formula (1), *u*_1_ and *θ*_1_ are obtained. And then, the position vector $r_{f}^{\left(M_{j}^{1}\right)}$ of $M_{j}^{1}\;(x_{j}^{1},y_{j}^{1},z_{j}^{1})$ and the position vector $r_{f}^{\left(M_{j}^{2}\right)}$ of $M_{j}^{2}\;(x_{j}^{2},y_{j}^{2},z_{j}^{2})$ are obtained. Through coordinate transformation, $r_{f}^{\left(M_{j}^{1}\right)}$ and $r_{f}^{\left(M_{j}^{2}\right)}$ is transformed into the coordinate system *S*_1_ and *S*_2_. Then, $r_{f}^{\left(M_{j}^{1}\right)}$ and $r_{f}^{\left(M_{j}^{2}\right)}$ can be obtained. In the coordinate system *S*_1_ and *S*_2_, the absolute velocity at the point $M_{j}^{1}$ of the pinion tooth surface and the absolute velocity at the point $M_{j}^{2}$ of the pinion tooth surface can be expressed as the following Eq (2) and Eq (3).

$$u_{1}^{\left(M_{j}^{1}\right)}=w_{1}\times r_{1}^{\left(M_{j}^{1}\right)}$$ (2)

$$u_{2}^{\left(M_{j}^{2}\right)}=w_{2}\times r_{2}^{\left(M_{j}^{2}\right)}$$ (3)

Here, *w*_1_ and *w*_2_ are the angular velocity vector. Through coordinate transformation, the velocity vector $u_{f}^{\left(M_{j}^{1}\right)}$ and $u_{f}^{\left(M_{j}^{2}\right)}$ at the point $M_{j}^{1}$ and $M_{j}^{2}$ are obtained. $u_{f}^{\left(M_{j}^{1}\right)}$ and $u_{f}^{\left(M_{j}^{2}\right)}$ are projected to the $n_{f}^{\left(M_{0}\right)}$ direction, respectively, to get $u_{fn}^{\left(M_{j}^{1}\right)}$ and $u_{fn}^{\left(M_{j}^{2}\right)}$. Neglecting the influence of the clearance of the tooth surface and the elastic deformation, the tangential velocity $u_{f}^{\left(M_{j}^{1}\right)}$, $u_{ft}^{\left(M_{j}^{2}\right)}$ and the relative sliding velocity $u_{12}^{\left(M_{j}\right)}$ at the discrete point *M*_*j*_ are expressed as follow

$$u_{ft}^{\left(M_{j}^{1}\right)}=u_{f}^{\left(M_{j}^{1}\right)}-u_{fn}^{\left(M_{j}^{1}\right)}$$ (4)

$$u_{ft}^{\left(M_{j}^{2}\right)}=u_{f}^{\left(M_{j}^{2}\right)}-u_{fn}^{\left(M_{j}^{2}\right)}$$ (5)

$$u_{12}^{\left(M_{j}^{}\right)}=u_{ft}^{\left(M_{j}^{1}\right)}-u_{ft}^{\left(M_{j}^{2}\right)}$$ (6)

The length of *M*_*0*_*M*_*j*_ changed with a certain step length until the relative sliding speed of all discrete points on all meshing lines is calculated. The normal load and load density at discrete points at arbitrary meshing positions are obtained by LTCA.

**Fig 4:**
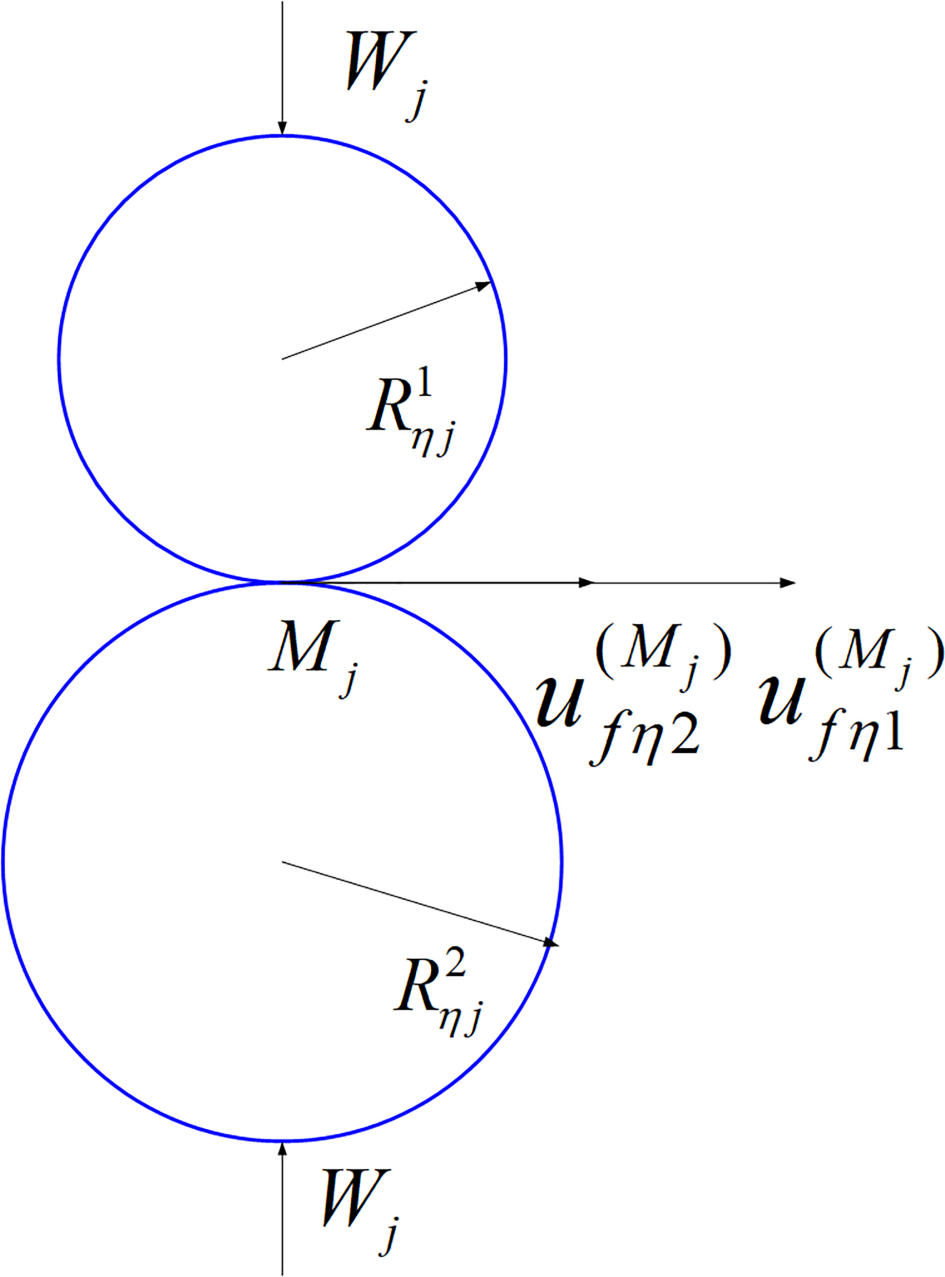
Equivalent cylindrical contact.

The actual lubrication state of gear is mixed Elastohydrodynamic lubrication both complete Elastohydrodynamic and boundary lubrication, the coefficient of sliding friction is an important factor affect the efficiency of gear drive. The lubrication of the gear in each of the discrete meshing lines is shown in **Fig** 4. Here, *W*_*j*_ is the normal load density. $R_{\eta j}^{1}$ and $R_{\eta j}^{2}$ are the equivalent radius. $u_{f\eta 1}^{M_{j}}$ and $u_{f\eta 2}^{M_{j}}$ are the movement speed at the contact point *M*_*j*_. Because the gear is in full elastohydrodynamic lubrication, the two contact surface is completely separated by a certain thickness of the lubricating oil film. Because of the viscous friction in the lubricating oil film, the friction force on the two contact surfaces along the moving direction is the integral of the shear stress in the whole lubricating layer in contact with the surface of the oil layer.

**Fig 5:**
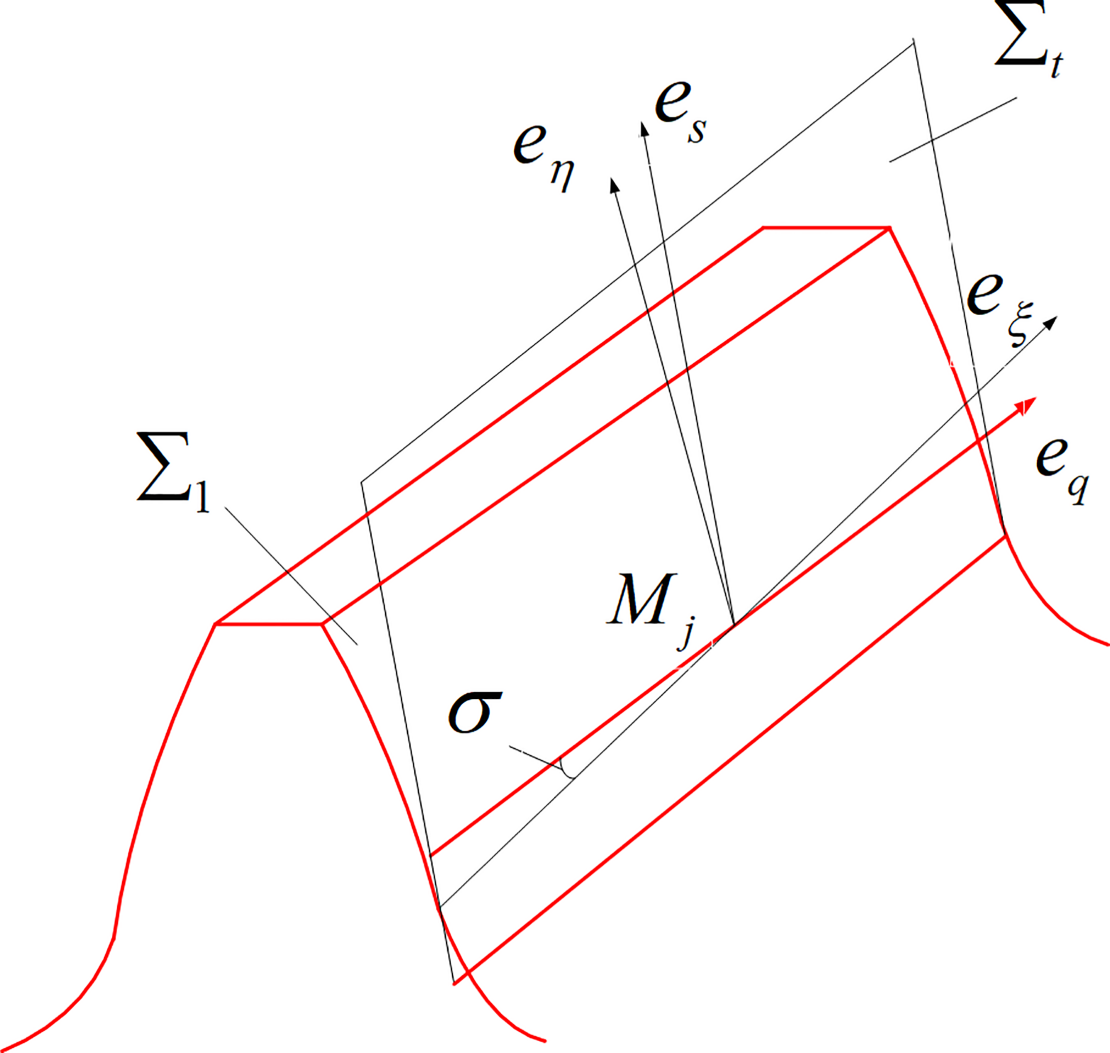
Tangent plane coordinates.

As shown in **Fig** 5, *e*_*ξ*_ and *e*_*η*_ are respectively the long axis and short axis of the contact ellipse on the tangent plane Σ_*t*_. *e*_*ξ*_ is the contact line direction.

*e*_*s*_ and *e*_*q*_ are two main directions at arbitrary discrete points *M*_*j*_ of the tooth surface Σ_1_ of the pinion in the contact line; The angle between the axis *e*_*ξ*_ and *e*_*q*_ is *σ*. The curvature of the tooth surface of the gear for the equivalent cylindrical curvature in the *e*_*η*_ direction at discrete points *M*_*j*_. According to the EULER formula, the normal curvature of the *e*_*η*_ direction at the discrete point *M*_*j*_ can be expressed as the following

$$K_{e\eta j}^{1}=K_{esj}^{1}\cos^{2}\sigma +K_{eqj}^{1}\sin^{2}\sigma$$ (7)

Here, $K_{enj}^{1}$ is the normal curvature at discrete points *M*_*j*_ in the *e*_*η*_ direction, the equivalent cylinder radius of pinion is $R_{\eta j}^{1}=1/K_{enj}^{1}$. $K_{esj}^{1}$ and $K_{eqj}^{1}$ are the main curvature. Due to the tooth surface curvature of face gear is very small [24], so it can be simplified to one plane, that is $R_{\eta j}^{2}$ = ∞. Due to the using one-dimensional Elastohydrodynamic lubrication model, the relative motion of the two tooth surface in the *ξ* direction is ignored. The tangential velocity of the two cylindrical surface are $u_{ft}^{\left(M_{j}^{1}\right)}$ and $u_{ft}^{\left(M_{j}^{2}\right)}$. The projection of this two tangential velocity in *e*_*η*_ direction are $u_{f\eta }^{\left(M_{j}^{1}\right)}$ and $u_{f\eta }^{\left(M_{j}^{2}\right)}$.

The distribution of oil film pressure and the thickness distribution of the film can be obtained by solving Reynolds equation, viscosity equation, density equation, energy balance equation and load equation. The distribution of the shear stress in the oil film can be obtained according to the distribution of oil film pressure, the thickness distribution of the film and the equation of the Ree-Eyring fluid. According to the distribution of shear stress obtained, the friction and friction coefficient between the two contact surfaces can be obtained. Yang's theory [25] of non Newton time-varying micro thermal elastohydrodynamic theory takes into account the effect of thermal effect, surface roughness, time-varying effect and non Newton flow on elastohydrodynamic lubrication. In order to have better convergence and faster computation speed, a steady thermal elastohydrodynamic model is established to solve the sliding friction coefficient. It is assumed that the tooth surface is completely smooth, that is, the gear is in complete elastohydrodynamic lubrication. The constitutive equation of Ree-Eyring fluid is

$$\tau =\nu^{*}\frac{\partial u}{\partial z}$$ (8)

**Fig 6:**
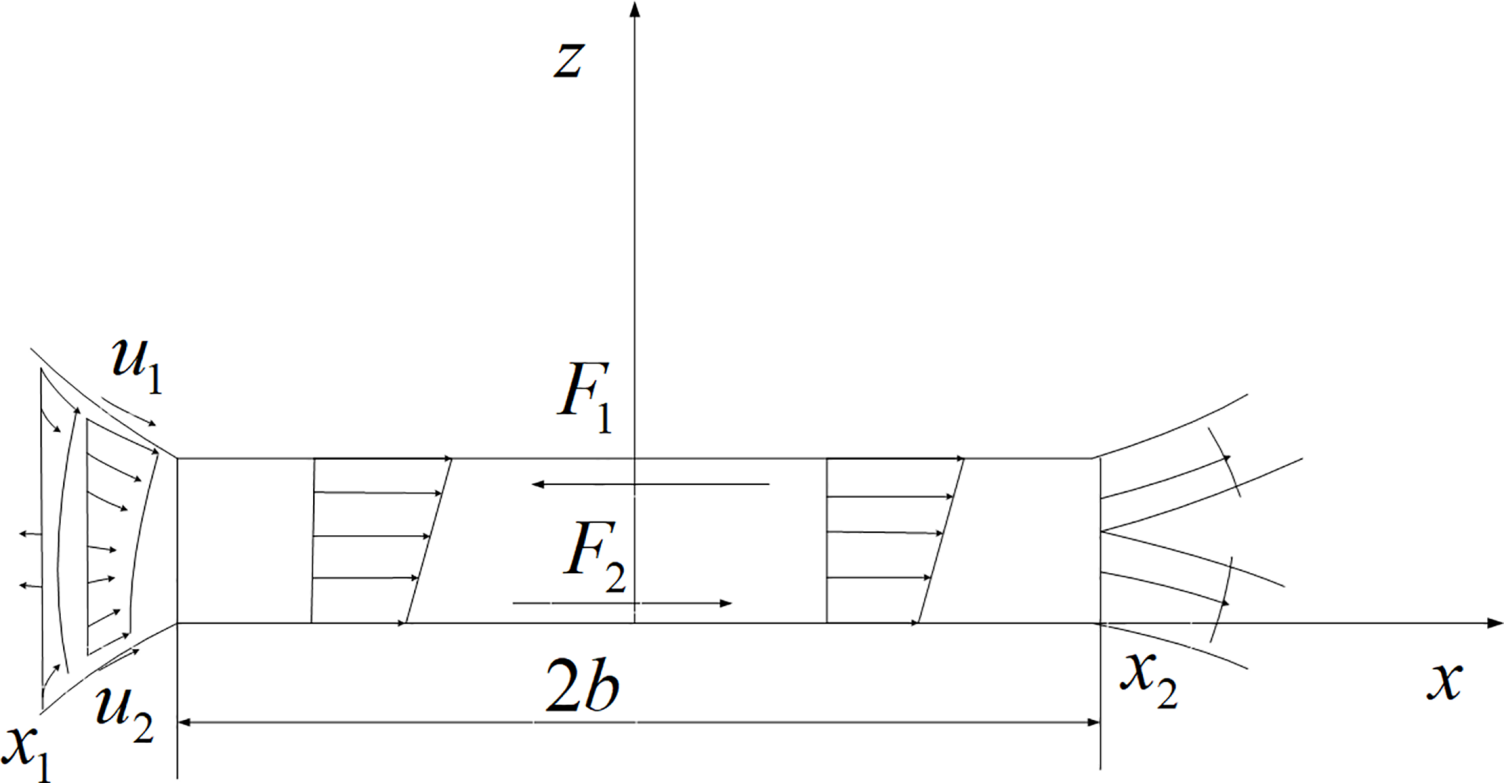
The velocity distribution and friction.

Here, *τ* is oil film shear stress (Pa), *u* is flow velocity of lubricating oil along *x* direction (m/s, as shown in **Fig** 6), v^*^ is equivalent viscosity (Pa·s) of lubricating oil, v^*^ = *v*(*τ/τ*_0_) *v* is apparent viscosity (Pa·s) of Ree-Eyring fluid, *τ*_0_ is shear stress (Pa) of Ree-Eyring fluid.

Reynolds equation is follow[25]

$$\frac{\partial }{\partial x}[{\left(\frac{\rho }{\nu }\right)}_{e}h^{3}\frac{\partial p}{\partial x}]=12U\frac{\partial (\rho^{*}h)}{dx}$$ (9)

Here, *p* is oil film pressure (Pa); *h* is the oil film thickness (m); *U* is the volume absorption rate (m/s), *U =* (*u*_1_+*u*_2_)/2, *u*_1_ and *u*_2_ are the surface velocity of two cylinder; ρ* and (*ρ*/*v*)_*e*_ is equivalent parameters relating to fluid viscosity *v* and density *ρ*. These parameters consist of the following differential equations

$$\left\{ \begin{array}{l} {\left(\frac{\rho }{\nu }\right)}_{e}=12(\nu_{e}\rho {'}_{e}/\nu {'}_{e}-\rho '{'}_{e})\\ \rho^{*}=[\rho {'}_{e}\nu_{e}(u_{1}-u_{2})+\rho_{e}u_{1}]/U\\ \rho_{e}=\frac{1}{h}\int_{0}^{h}\rho dz\\ \rho {'}_{e}=\frac{1}{h^{2}}\int_{0}^{h}\rho \int_{0}^{z}\frac{dz'}{\nu^{*}}dz\\ \rho '{'}_{e}=\frac{1}{h^{3}}\int_{0}^{h}\rho \int_{0}^{z}\frac{z'dz'}{\nu^{*}}dz\\ \frac{1}{\nu_{e}}=\frac{1}{h}\int_{0}^{h}\frac{dz}{\nu^{*}};\frac{1}{\nu {'}_{e}}=\frac{1}{h^{2}}\int_{0}^{h}\frac{zdz}{\nu^{*}} \end{array}\right.$$ (10)

The boundary conditions for the differential equations are *p*(*x*_*in*_) = 0,*p*(*x*_*out*_) = 0,*p*(*x*)>0,(*x*_*in*_<*x*<*x*_*out*_)

The oil film thickness equation is

$$\begin{array}{l} h(x)=h_{0}+\frac{x^{2}}{2R}+\delta (x)\\ \to \delta (x)=-\frac{2}{\pi E'}\int_{x_{in}}^{x_{out}}p(s)\ln{\left(x-s\right)}^{2}ds \end{array}$$ (11)

Here, *h*_0_ is the central film thickness (m) of the cylinder without deformation, *R* is the equivalent radius (m) of the cylinder; And, *δ*(x) is the sum of the normal elastic deformation of the two surface (m), *E*' is equivalent elastic modulus (Pa)

Energy equation of oil film is

$$c(\rho u\frac{\partial T}{\partial x}+\rho w\frac{\partial T}{\partial z})-k\frac{\partial^{2}T}{\partial z^{2}}=-u\frac{\partial p}{\partial x}\frac{T}{\rho }\frac{\partial \rho }{\partial T}+\tau \frac{\partial u}{\partial z}$$ (12)

Here, *c* is the specific heat capacity (J/(kg·K)); *T* is the temperature (K); *ρ* is the density (Kg/m^3^); *u* is the velocity in the *x* direction (m/s); *w* is the velocity in the z direction (m/s); *k* is the heat transfer coefficient of lubricating oil.

Two the energy equation of the cylinder is

$$\{\begin{array}{c} c_{1}\rho_{1}(u_{1}\frac{\partial T}{\partial x})=k_{1}\frac{\partial^{2}T}{\partial z_{1}^{2}}\\ c_{2}\rho_{2}(u_{2}\frac{\partial T}{\partial x})=k_{2}\frac{\partial^{2}T}{\partial z_{2}^{2}} \end{array}$$ (13)

Here, *c*_1_ and *c*_2_ are the specific heat capacity of materials of pinion and gear (J/(kg·K)); *ρ*_1_ and *ρ*_2_ are the density of materials of pinion and gear (Kg/m^3^); k_1_ and *k*_2_ is heat conduction coefficient of materials of pinion and gear; z_1_ and z_2_ are the space coordinates of materials of pinion and gear, that is the z coordinate of **Fig** 6.

Solid temperature and liquid temperature are continuous at the solid-liquid interface, so it can be expressed as follows.

$$\{\begin{array}{c} k\frac{\partial T}{\partial z}{|}_{z=0}=k_{a}\frac{\partial T}{\partial z_{a}}{|}_{z_{a}=0}\\ k\frac{\partial T}{\partial z}{|}_{z=h}=k_{b}\frac{\partial T}{\partial z_{b}}{|}_{z_{b}=0} \end{array}$$ (14)

Here, The boundary conditions of the oil film upstream temperature are *T*(*x*_*in*_,*z*) = *T*_0_ (*u*(*x*_*in*_,*z*)> = 0).

The temperature boundary conditions are *T|*_*z*1 = -*d*_ = *T*_0_, *T|*_*z*2 = *d*_ = *T*_0_, *d* is the temperature depth (m), *d* = 3.15b, *b* is the half width of the contact area of the Hertz (m, as shown in **Fig** 6).

Viscosity equation and density equation are respectively:

$$\begin{array}{l} \left\{ \begin{array}{l} \nu =\nu_{0}\exp\{(\ln\nu_{0}+9.67)\\ \left[{\left(1+5.1\times 10^{-9}p\right)}^{z_{0}}{\left(\frac{T-138}{T_{0}-138}\right)}^{-s_{0}}-1]\right\}\\ \rho =\rho_{0}[1+\frac{0.6\times 10^{-9}p}{1+1.7\times 10^{-9}p}\\ -0.00065(T-T_{0})] \end{array}\right.\\ \to z_{0}=\alpha /[5.1\times 10^{-9}\times (\ln\nu_{0}+9.67)]\\ \to s_{0}=\beta /[(\ln\nu_{0}+9.67)/(T_{0}-138)] \end{array}$$ (15)

Here, *v*_0_ and ρ_0_ are the environment viscosity (Pa·s) and environmental density (kg/m^3^),*α* and *β* are the viscosity coefficient (Pa^-1^) and viscosity temperature coefficient (*K*^-1^);*T*_0_ is the ambient temperature (K).

The load equation can be further obtained, which is expressed as follows

$$W=\int_{x_{\mathrm{in}}}^{x_{\mathrm{out}}}pdx$$ (16)

Here, *W* is the applied load (N).

As shown in **Fig** 6, the frictional force on the contact surface and the velocity distribution of the lubricating oil.

Here, *x*_1_ and *x*_2_ is the starting positiom and final positiom of the oil film, *F*_1_ and *F*_2_ are the friction force on the two surfaces. The rolling friction is mainly generated in the entrance area (*x*_1_,-*b*), the sliding friction is mainly generated in the contact area (-*b*,*b*), so the sliding friction of the two surface is

$$F_{s}=(F_{1}+F_{2})/2\left\{ \begin{array}{l} \to F_{1}=\int_{-b}^{b}\tau {|}_{z=0}dx\\ \to F_{2}=\int_{-b}^{b}\tau {|}_{z=h}dx \end{array}\right.$$ (17)

The sliding friction coefficient can be expressed as

$$\mu_{j}=\frac{F_{s}}{W}$$ (18)

The above mathematical model is dimensionless, the multi grid method is used to solve the pressure. The temperature is solved by the column by line scanning technique, and the complete numerical solution is obtained through repeated iteration of pressure and temperature. At discrete points *M*_*j*_, giving $R=R_{nj}^{1}R_{nj}^{2}/(R_{nj}^{1}+R_{nj}^{2})$, $u_{1}=u_{f\eta }^{\left(M_{j}^{1}\right)}$ and $u_{2}=u_{f\eta }^{\left(M_{j}^{2}\right)}$, by solving the instantaneous sliding friction coefficient at the discrete points *M*_*j*_, the instantaneous sliding friction coefficient at each discrete point in each meshing position can be obtained by the same method.

The instantaneous Sliding friction coefficient is solving at the discrete point *M*_*j*_ by solving the above equations. By using the same method, the instantaneous Sliding friction coefficient of each discrete point in each meshing position can be obtained. The calculated value of Sliding friction coefficient based on TEHL is very close to the experimental value, but the method has no analytical solution, and the numerical calculation time is very long and the algorithm is very complex. Therefore, here the semi-empirical formula has been widely adopted by Benedict- Kelly[18].

It is proved that under the condition of mixed elastohydrodynamic lubrication, the sliding friction factor increases with the relative sliding velocity at a relatively low relative sliding speed, and decreases with the increase of relative sliding velocity when the relative sliding velocity is higher, and the sliding friction coefficient is 0 at the node. But in the vicinity of the node, the Benedict -Kelly model increases with the decrease of relative sliding velocity when the relative sliding velocity is low, so the formula is improved. [18,24].

$$\begin{array}{l} f_{j}(\varphi_{i})=\{\begin{array}{c} f_{0.06}^{M_{j}}\frac{s_{r}^{M_{j}}(\varphi_{i})}{0.06}\begin{array}{cc} , & \end{array}(s_{r}^{M_{j}}(\varphi_{i})<0.06)\\ 0.0127\log\frac{59.32W_{j}(\varphi_{i})}{\mu_{0}s_{r}^{M_{j}}(\varphi_{i}){\left[v_{12}^{M_{j}}(\varphi_{i})\right]}^{3}}\begin{array}{cc} , & \left(s_{r}^{M_{j}}(\varphi_{i})\geq 0.06\right) \end{array} \end{array}\\ s_{r}^{M_{j}}(\varphi_{i})=2|u_{12}^{M_{j}}(\varphi_{i})/v_{12}^{M_{j}}(\varphi_{i})|,\;v_{12}^{M_{j}}(\varphi_{i})=u_{ft}^{\left(M_{j}^{1}\right)}+u_{ft}^{\left(M_{j}^{2}\right)} \end{array}$$ (19)

$$f_{0.06}^{M_{j}}=0.0127\log\frac{59.32W_{j}(\varphi_{i})}{\mu_{0}0.06{\left[v_{12}^{M_{j}}(\varphi_{i})\right]}^{3}}$$ (20)

Here, *f*_*j*_(*φ*_*i*_) is the instantaneous Sliding friction coefficient, $s_{r}^{M_{j}}(\varphi_{i})$ is slip ratio, $u_{12}^{M_{j}}(\varphi_{i})$ and $v_{12}^{M_{j}}(\varphi_{i})$ is Relative sliding velocity (m/s) and relative rolling speed (m/s); *W*_*j*_(*φ*_*i*_) is normal load density (N/m); *μ*_0_ is dynamic viscosity (Pa·s) of lubricating oil; $f_{0.06}^{M_{j}}$ is the instantaneous Sliding friction coefficient of slip ratio 0.06 the same with the load density and the same rolling speed condition.

**Fig 7:**
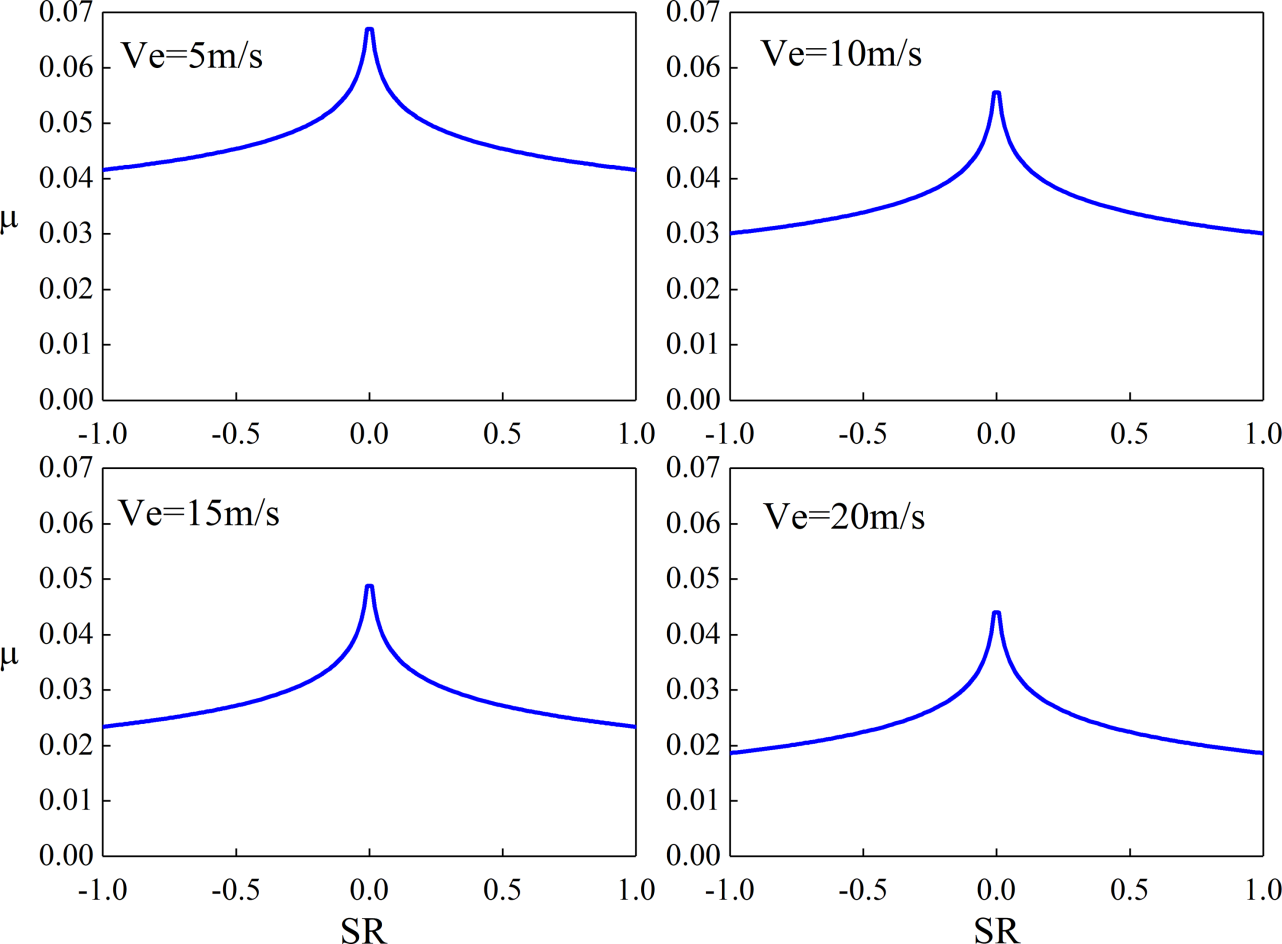
Kelly Benedict formula calculated value (R = 0.020638m, Ph = 1.0 GPa).

**Fig 8:**
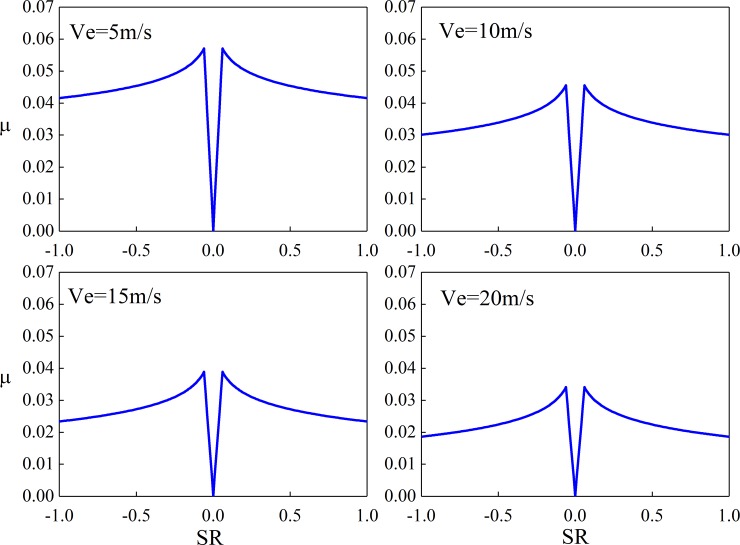
The calculated values of the improved formula (R = 0.020638m, Ph = 1.0 GPa).

**Fig** 7 and **Fig** 8 are the calculated value of Kelly Benedict formula and the improved Kelly Benedict formula. Here, R is the radius of the equivalent cylinder, Ph is the maximum Hertz pressure, Ve is the speed of entrainment, and its value is half of relative rolling speed, μ is the instantaneous Sliding friction coefficient.

As can be seen from **Fig** 7 and **Fig** 8, the results of Benedict and Kelly calculation are accurate when the relative sliding speed is high, but when the sliding speed is low, the error of the Benedict and Kelly calculation result is larger. The revised Benedict and Kelly formula can effectively reduce the calculation error of friction coefficient when sliding speed is low. The larger the relative sliding speed, the smaller the sliding friction coefficient.

**Fig 9:**
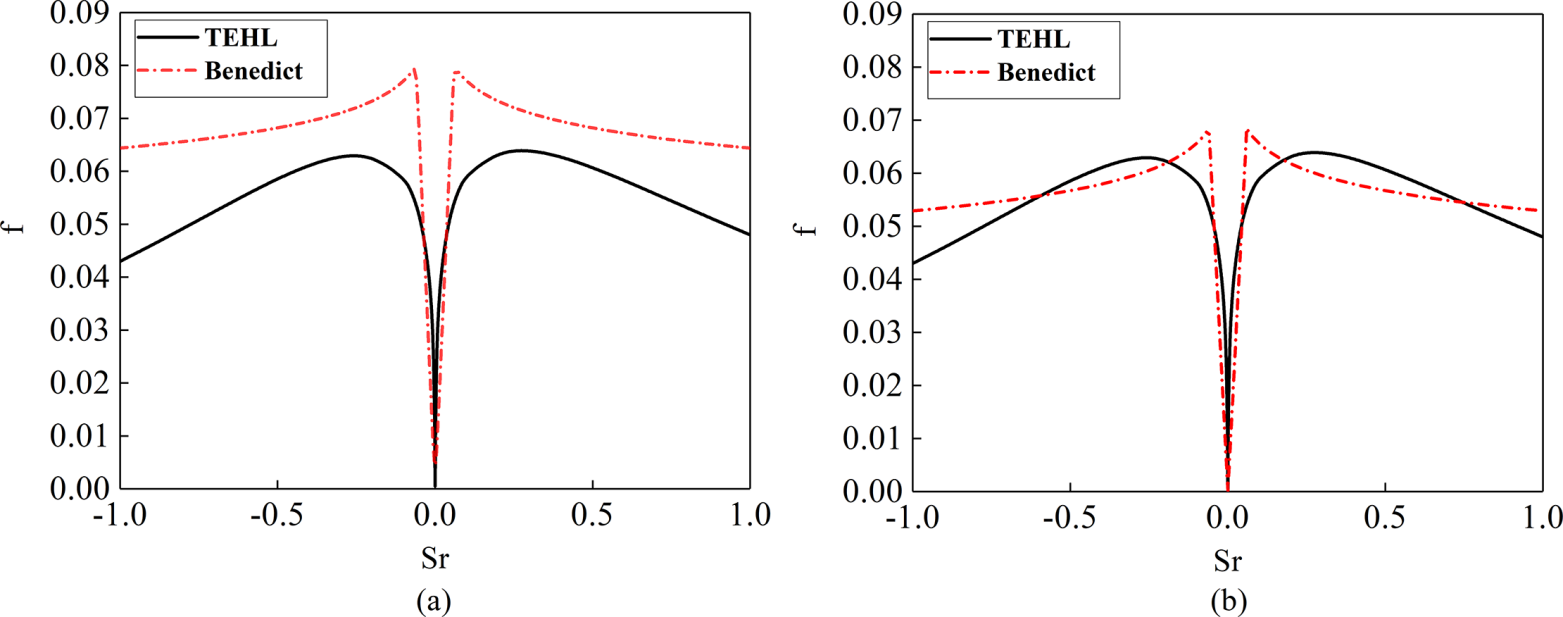
Comparison between TEHL theory and improved formula. (a) Normal load density is 1000000N/m; Relative rolling speed is 1.5m/s; Equivalent cylindrical radius is 0.05m. (b) Normal load density is 1000000N/m; Relative rolling speed is 3m/s; Equivalent cylindrical radius is 0.08m.

The thermal elastohydrodynamic theory is compared with the improved Benedict and Kelly formula, the results is shown in **Fig** 9. The working conditions of **Fig** 9(A) are as follows: normal load density is 1000000N/m, relative rolling speed is 1.5m/s, and the equivalent cylinder radius is 0.05m. The working conditions of **Fig** 9(B) are as follows: normal load density is 1000000N/m, relative rolling speed is 3m/s, and the equivalent cylinder radius is 0.08m.

In order to facilitate calculation convergence, the influence of surface roughness is not considered, and the lubrication condition of gear is assumed to be complete elastohydrodynamic lubrication. As can be seen from **Fig 9(A) and 9(B)**, the sliding friction coefficient obtained by the improved formula is close to the value obtained by the theory of thermal elastohydrodynamic theory. Due to the lack of surface roughness in elastohydrodynamic lubrication and the assumption that the lubrication state of the gear is complete elastohydrodynamic lubrication, the sliding friction system obtained by the theory of elastohydrodynamic lubrication is smaller.

## Calculation of meshing efficiency

After the instantaneous Sliding friction coefficient *f*_*j*_(*φ*_*i*_) of discrete point *M*_*j*_ at the engagement point *φ*_*i*_ is obtained, the sliding friction at the discrete point *M*_*j*_ is

$$F_{fj}(\varphi_{i})=f_{j}(\varphi_{i})F_{nj}(\varphi_{i})$$ (21)

Here, *F*_*nj*_(*φ*_*i*_) is a normal load of engagement position *φ*_*i*_ at discrete point *M*_*j*_

The sliding friction power loss of the tooth surface is obtained by the general dynamic power calculation method at engagement position *φ*_*i*_

$$P_{f}(\varphi_{i})=\sum_{j=1}^{n(i)}F_{fj}(\varphi_{i})|u_{12}^{\left(M_{j}\right)}(\varphi_{i})|$$ (22)

Here, $u_{12}^{M_{j}}(\varphi_{i})$ is the relative sliding speed of the two tooth surface, *n*(*i*) is a number of discrete points.

The instantaneous Meshing Efficiency of the gear in meshing position is obtained by only considering the sliding friction power loss.

$$\eta (\varphi_{i})=1-\frac{P_{f}(\varphi_{i})}{P_{in}}$$ (23)

Here, *P*_*in*_ is the gear input power.

By the above calculation, the instantaneous sliding friction power loss *P*_*f*_(*φ*_1_), *P*_*f*_(*φ*_2_),…, *P*_*f*_(*φ*_m_) is obtained corresponding to the engagement positions *φ*_1_, *φ*_2_,…, *φ*_m_ in the mesh period. Relationship between ferry angle and instantaneous efficiency was obtained by least square method

$$P_{f}(\varphi )=a_{1}+a_{2}\varphi '+a_{3}{\left(\varphi '\right)}^{2}+a_{4}{\left(\varphi '\right)}^{3}(\varphi '=\varphi -\sum_{i=1}^{m}\varphi_{i}/m)$$ (24)

Here, *a*_1_, *a*_2_, *a*_3_, *a*_4_ is the coefficient of polynomial.

The mean meshing efficiency of gear is obtained by calculating the mean value of the gear rotation angle in the meshing period. It can be expressed as the following formula:

$$\bar{P_{f}}=\frac{1}{\left(\varphi_{m}-\varphi_{1}\right)}\int_{\varphi_{1}}^{\varphi_{m}}P_{f}(\varphi )d\varphi$$ (25)

## Calculation of churning oil loss and windage loss

If the lubrication method is oil-immersed lubrication, the main loss of gear efficiency is churning oil loss (*P*_*ch*_) and sliding friction power loss (*P*_*f*_). The crush loss of the lubricating oil in the gear meshing zone is omitted, and the churning oil loss (*P*_*ch*_) of each gear is calculated separately regardless of the efficiency loss of the shaft.

The empirical formula of Boness is used to calculate the churning oil loss of gears [26].

$$P_{ch}=\frac{1}{2}\rho \omega^{3}S_{m}r_{p}{}^{3}C_{m}$$ (26)

$$\{\begin{array}{c} C_{m}=20/R_{e}\begin{array}{cc} , & R_{e}<2000 \end{array}\\ C_{m}=8.6\times 10^{-4}R_{e}{}^{1/3}\begin{array}{cc} , & 2000<R_{e}<100000 \end{array}\\ C_{m}=5\times 10^{8}/R_{e}{}^{2}\begin{array}{cc} , & 100000<R_{e} \end{array} \end{array}$$ (27)

$$S_{m}=r_{p}(2b\arccos(1-\frac{h_{sub}}{r_{p}}))+\frac{4Zh_{dent}b\arccos(1-\frac{h_{sub}}{r_{p}})}{2\pi \cos\alpha }+r_{p}{}^{2}(2\arccos(1-\frac{h_{sub}}{r_{p}})-\sin(2\arccos(1-\frac{h_{sub}}{r_{p}})))$$ (28)

$$R_{e}=\frac{\omega r_{p}{}^{2}}{\kappa }$$ (29)

Here, *P*_*ch*_ is gear’s churning oil loss (W), *ρ* is lubricating oil density (kg/m^3^), *ω* is gear’s angular velocity (rad/s), *S*_*m*_ is the gear’s wet surface area (m^2^), *r*_*p*_ is gear’s base circle radius (m), *C*_*m*_ is the dimensionless drag coefficient; *R*_*e*_ is Reynolds number, *b* is the tooth width (m), *h*_*sub*_ is gear immersion depth (m), *Z* is the number of teeth, *h*_*dent*_ is the tooth is high (m), *α* is the pressure angle (°); *κ* is lubricating oil viscosity (m/s^2^).

If the lubrication method is spray lubrication, the main loss of gears is windage loss (*P*_*win*_) and sliding friction power loss (*P*_*f*_). The method for calculating the windage loss of gears uses the empirical formula of Y.Diab. [27], the formula is as follows.

$$P_{win}=\frac{1}{2}C_{t}\rho_{win}\omega^{2}r_{p}^{5}$$ (30)

$$C_{t}=60R_{e}{}^{-0.25}{\left(\frac{b}{r_{p}}\right)}^{0.8}Z^{-0.4}$$ (31)

Here, *P*_*win*_ is gear windage loss, *C*_*t*_ is the dimensionless drag torque coefficient, *ρ*_*win*_ is the density of the fluid. Ω is the angular velocity, *r*_*p*_ is the pitch radius, *Re* is the reynolds number, *b* is the tooth width, *Z* is the etooth number.

Because of the proportion of the sliding friction power loss (*P*_*f*_) is very small, it is not considered. Therefore, the power loss (*P*_*l*_) of a single pair of gear pairs under oil-immersed and spray-lubricated conditions is

$$\left\{ \begin{array}{l} P_{l}=\bar{P_{f}}+P_{ch1}+P_{ch2}\\ P_{l}=\bar{P_{f}}+P_{win1}+P_{win2} \end{array}\right.$$ (32)

## Power loss calculation of spur-face gear

Taking a pair of Spur-Face gear as an example, the Meshing Efficiency is calculated. And the parameters of the gear and the oil parameters are shown in Table 1 and Table 2, respectively.

**Table 1:** Gear parameters.

Gear Parameters	Values
Pinion teeth N_1_	25
Shape wheel teeth N_s_	28
Face gear teeth N_2_	160
Module of gear m/mm	6
Normal pressure angle α_0_/(°)	25°
Shaft angle γm/(°)	90°
Inside radius L_1_/mm	480
External radius L_2_/mm	560

**Table 2:** Lubricating oil parameters.

Parameters	Values
Lubricating oil environment viscosity *υ*_0_ / Pa∙s	0.927
Lubricating oil environment density*ρ*_0_ /Kg/m^3^	900
Lubricating oil pressure coefficient *α* /Pa^-1^	2.389×10^−8^
Lubricating oil viscS^#osity-temperature coefficient *β* /K^-1^	0.042
Lubricating oil specific heat capacity *c J*/(kg∙K)	2000
Lubricating oil heat transfer coefficient *k* W/(m^-1^.K^-1^)	0.14
The gear material density *ρ*1、*ρ*2 /kg/m^3^	7850
Gear heat capacity *c*1、*c*2 *J*/(kg^-1^.K^-1^)	470
Gear heat transfer coefficient *k*1、*k*2 W/(m^-1^.K^-1^)	46
Equivalent elastic modulus Eʹ/Pa	227×10^9^
Environment temperature *T*_0_ /*K*	313
Characteristics of the shear stress τ_0_ /Pa	1.0×10^6^

**Fig 10:**
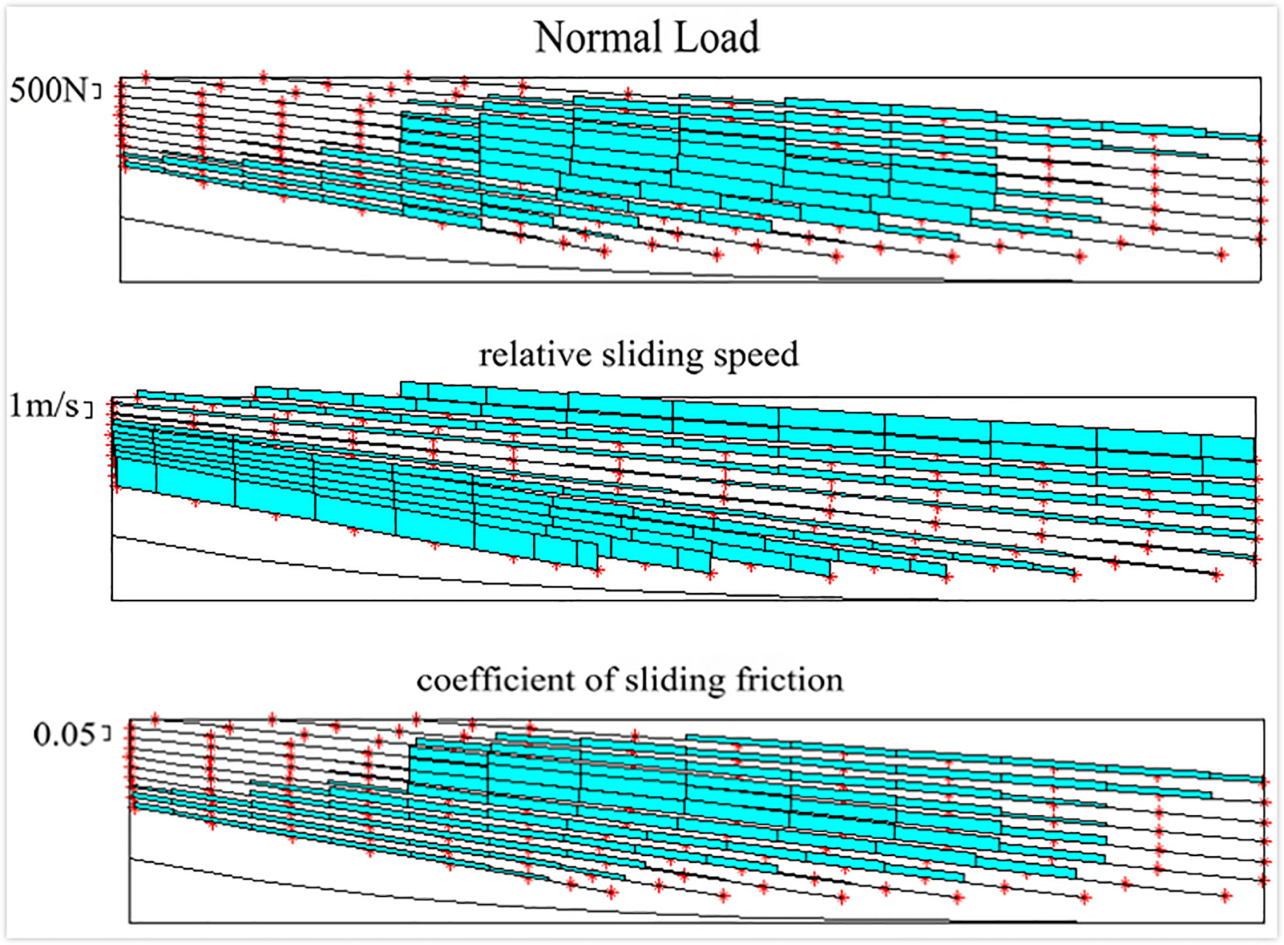
Normal load, relative sliding velocity and sliding friction coefficient. (a) Normal load. (b) Relative sliding speed. (c) Sliding friction coefficient.

**Fig** 10 is the normal load, relative sliding speed and Sliding friction coefficient of each discrete points on meshing line:

It can be seen from **Fig** 10 that the relative slip velocity near the nodal line is very small, and the relative slip velocity is larger at the farther away from the nodal line; the Sliding friction coefficients of the discrete points on different meshing lines are different, and the Sliding friction coefficients of different discrete points on the same meshing line are also different. The Meshing Efficiency of gears in different positions is different.

**Fig 11:**
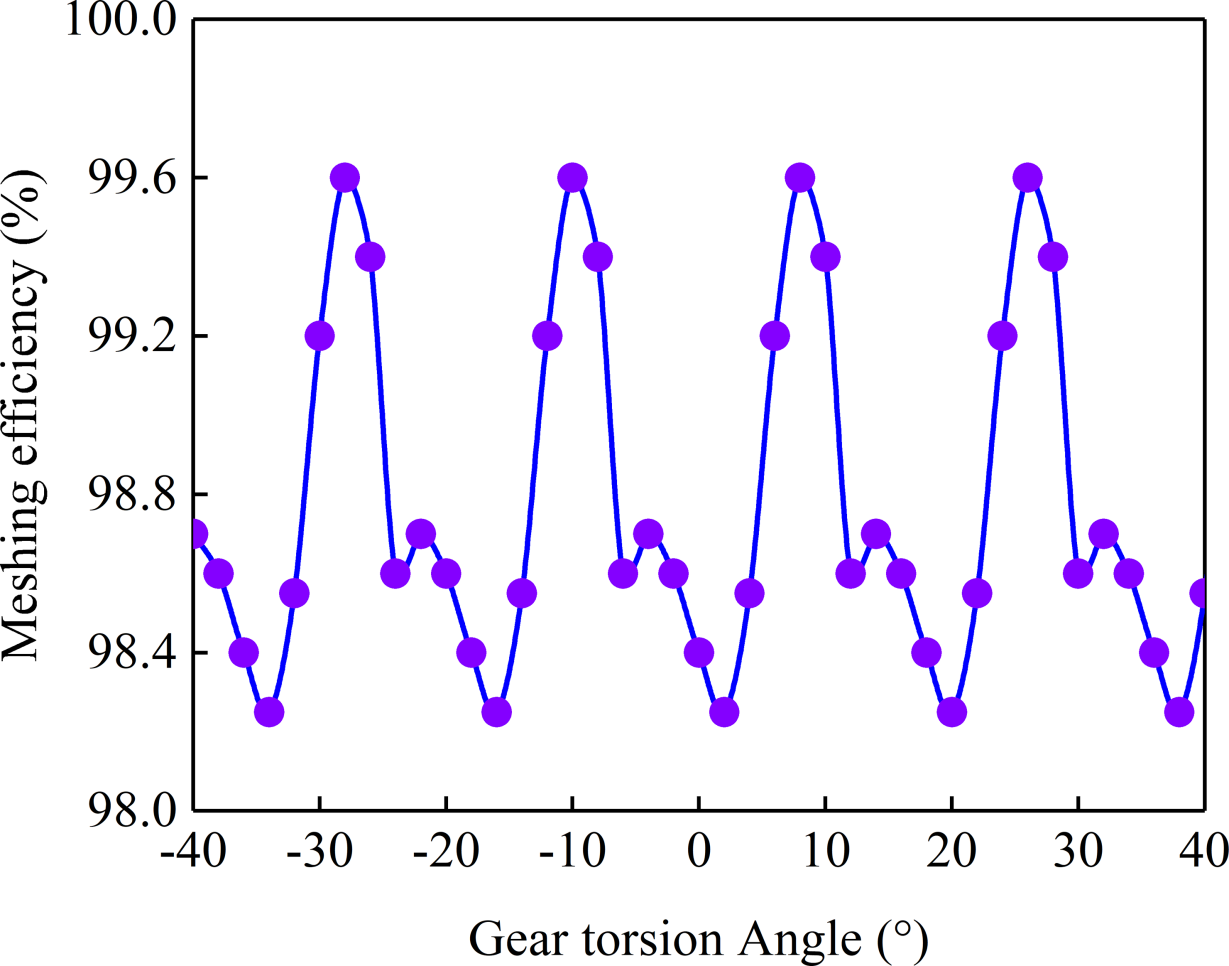
Meshing efficiency curve.

**Fig** 11 is the change curve of the Meshing Efficiency of the gear with the pinion angle, in which the turning angle of the pinion is a cycle. The input power is 73.3 kw and the input speed is 1000 r/min. It can be seen from **Fig** 9, the average Meshing Efficiency of the gear is 0.98808.

As can be seen from **Fig** 11, the relative sliding velocity near the nodal line is very small. The relative sliding velocity of the point on the meshing line, which is farther away from the segment line, is larger, the sliding friction coefficient of the discrete points on the different meshing lines is different, and the sliding friction coefficient of the different discrete points on the same meshing line is also different. The meshing efficiency of gears in different positions is different.

**Fig 12:**
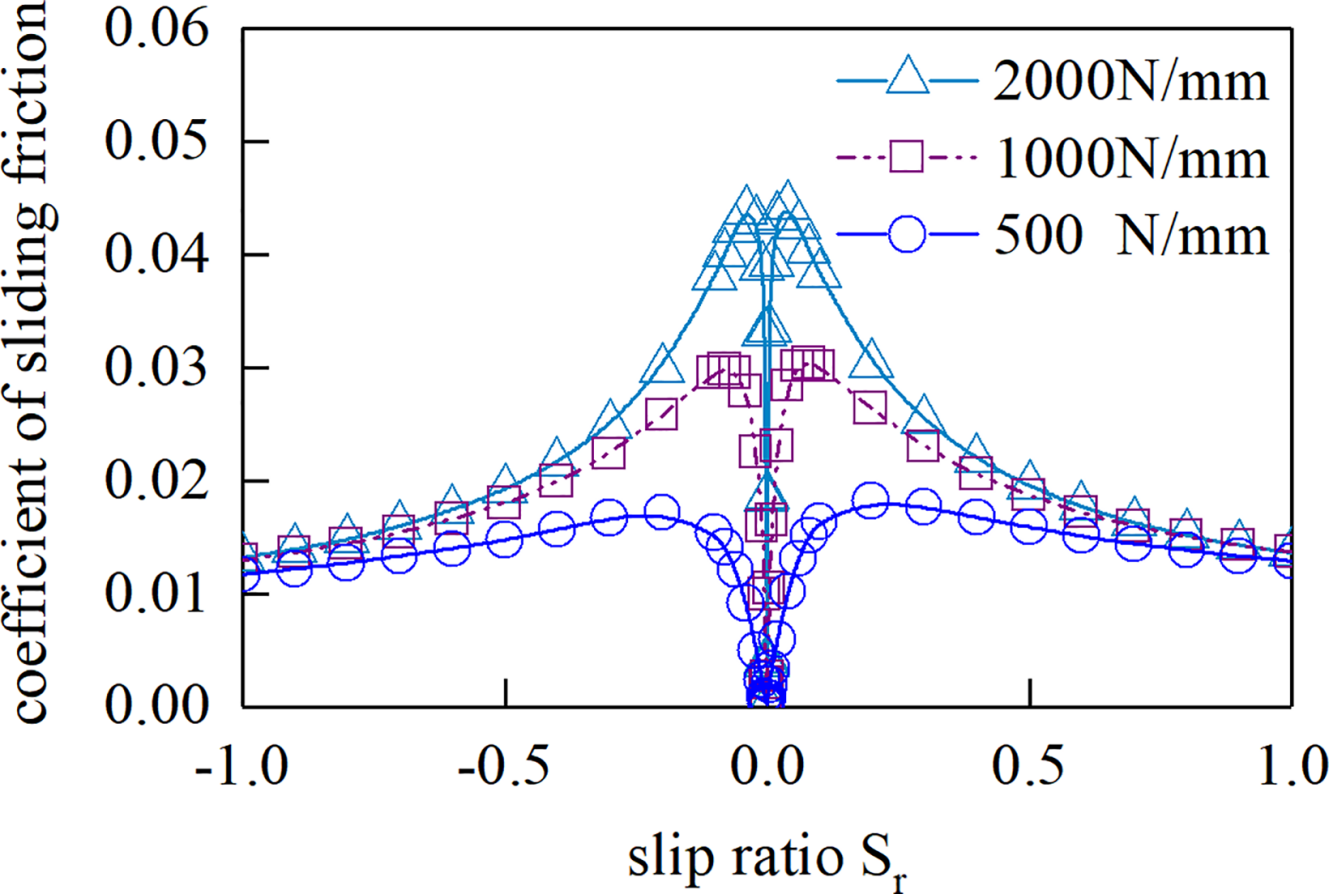
Sliding friction coefficient(*U* = 3.0 m/s).

**Fig 13:**
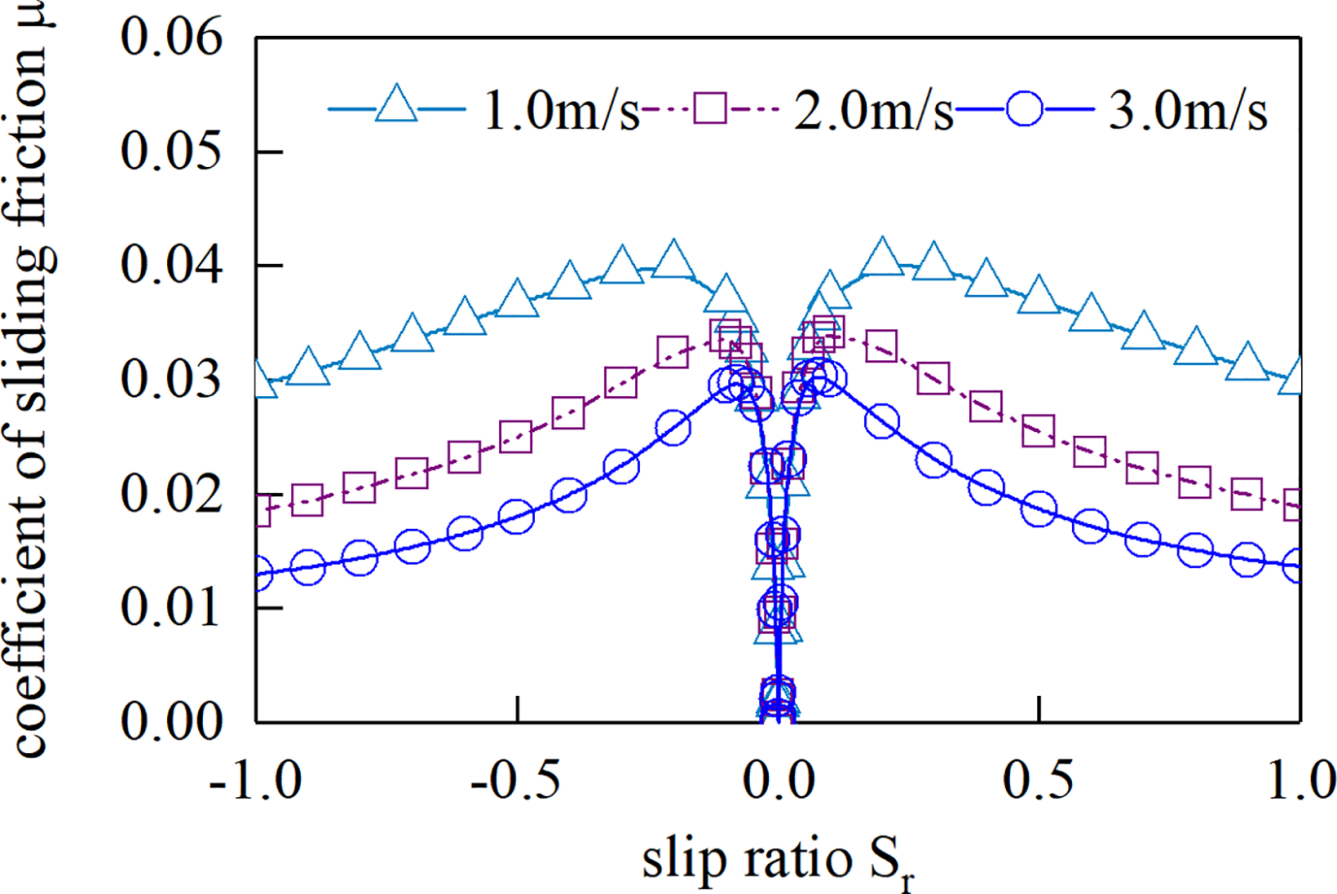
Sliding friction coefficient(*W* = 1 000 N/mm).

**Fig** 12 shows the sliding friction coefficient of different slip rates of a unit length cylinder and plane under different load densities. **Fig** 13 shows the sliding friction coefficient of different slip rates at different suction speeds. Here, the cylindrical radius is 0.08 m, the slip ratio is *S*_r_, *S*_r_ = 2(*u*_1_-*u*_2_)/(*u*_1_+*u*_2_).

It can be seen from **Fig** 12 and **Fig** 13 that the Sliding friction coefficient increases first and then decreases with the decrease of the sliding ratio, and the Sliding friction coefficient is zero at the pure rolling point. The relative Sliding friction coefficient increases with the increase of the normal load density, and decreases with the increase of the gear entrainment speed.

**Fig 14:**
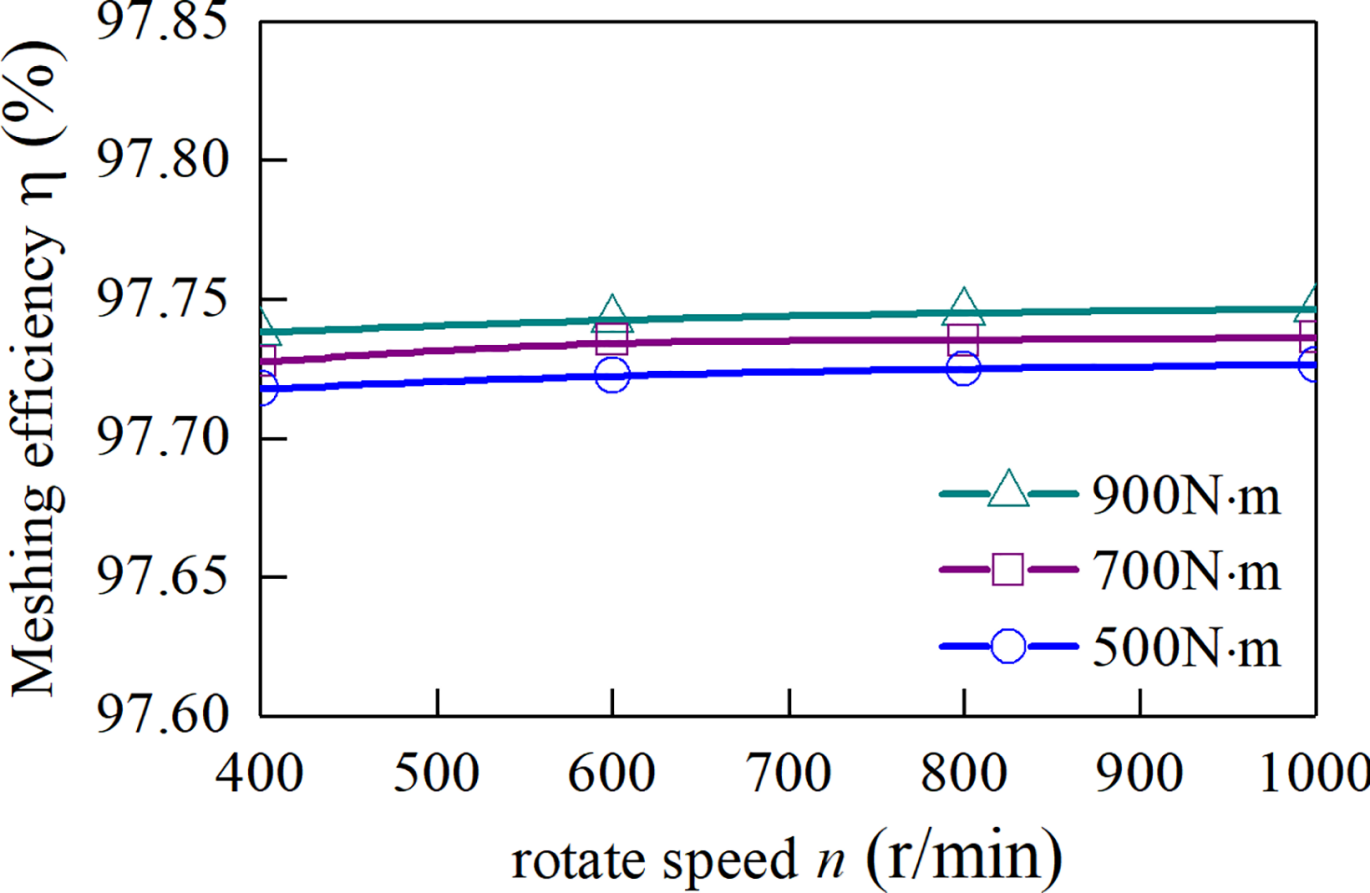
Average gear meshing efficiency with fixed friction coefficient conditions.

**Fig 15:**
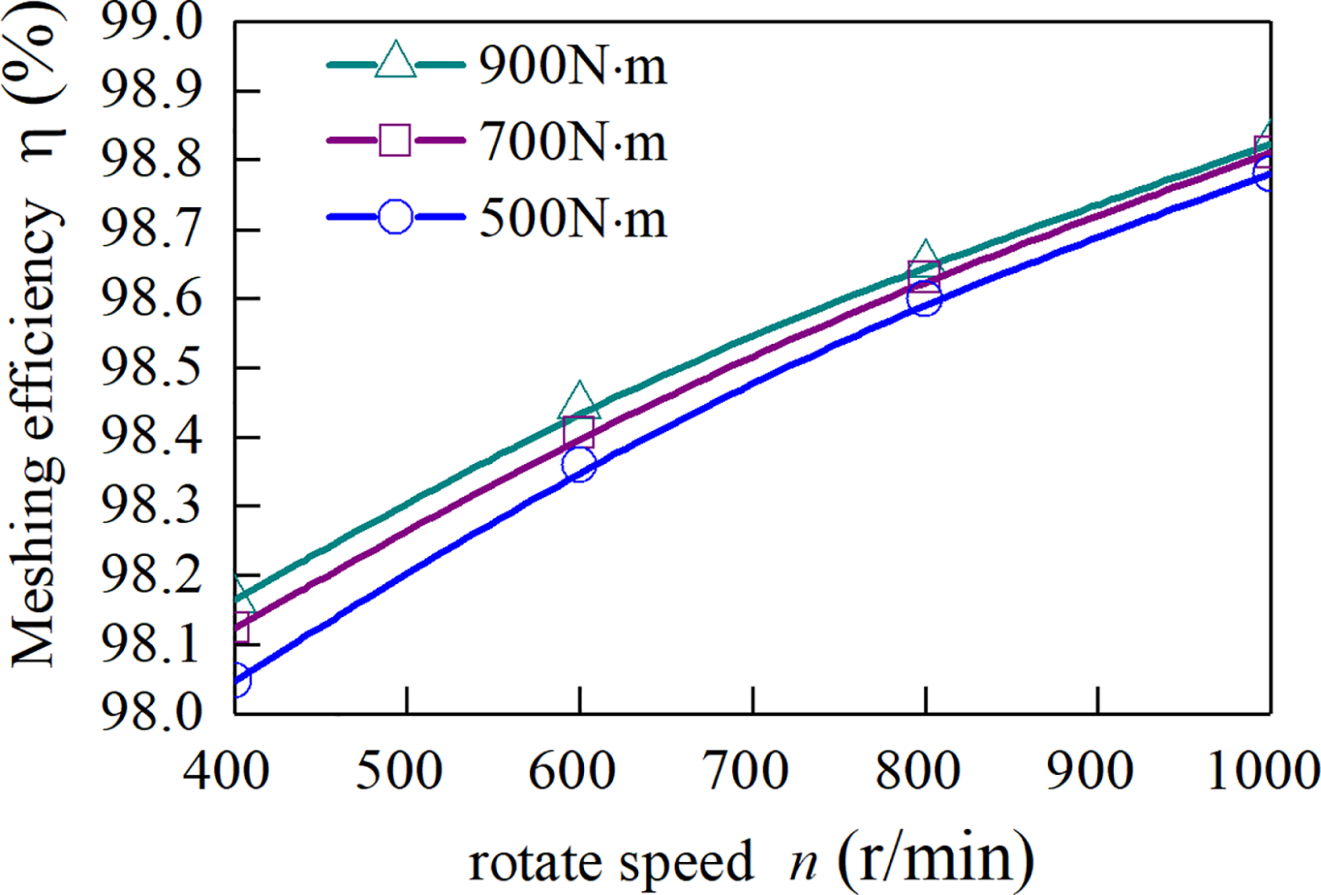
Average gear meshing efficiency.

When the friction coefficient is a fixed value of 0.1, the curves of the meshing efficiency of the spur-face gear with the speed of three sets of different input torque are obtained, as shown in **Fig** 14. It can be seen from **Fig** 14 that when the coefficient of friction is assumed to be a fixed value, the meshing efficiency of gears is not obvious with the change of speed and torque. **Fig** 15 is the change of the Meshing Efficiency of the Spur-Face gear with the rotation speed under the three input torques obtained by the above algorithm. It can be seen from **Fig** 15 that the Meshing Efficiency increases with the increase of rotation speed and decreases with the increase of torque. It shows that the influence of speed and torque on Meshing Efficiency is mainly achieved by friction coefficient.

## Analysis

**Fig 16:**
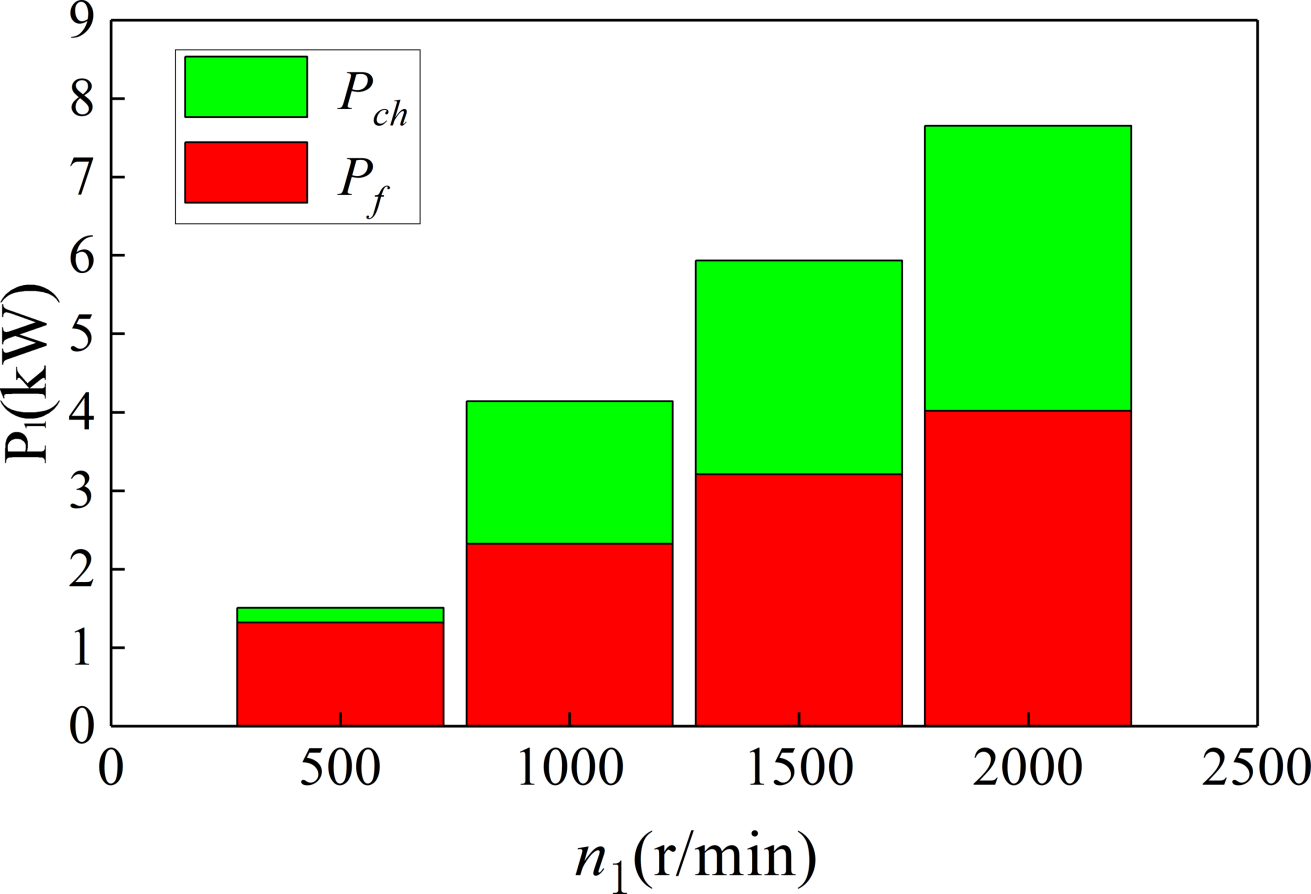
The power loss under the oil-immersed lubrication (*T*_2_ = 19950N·m).

**Fig 17:**
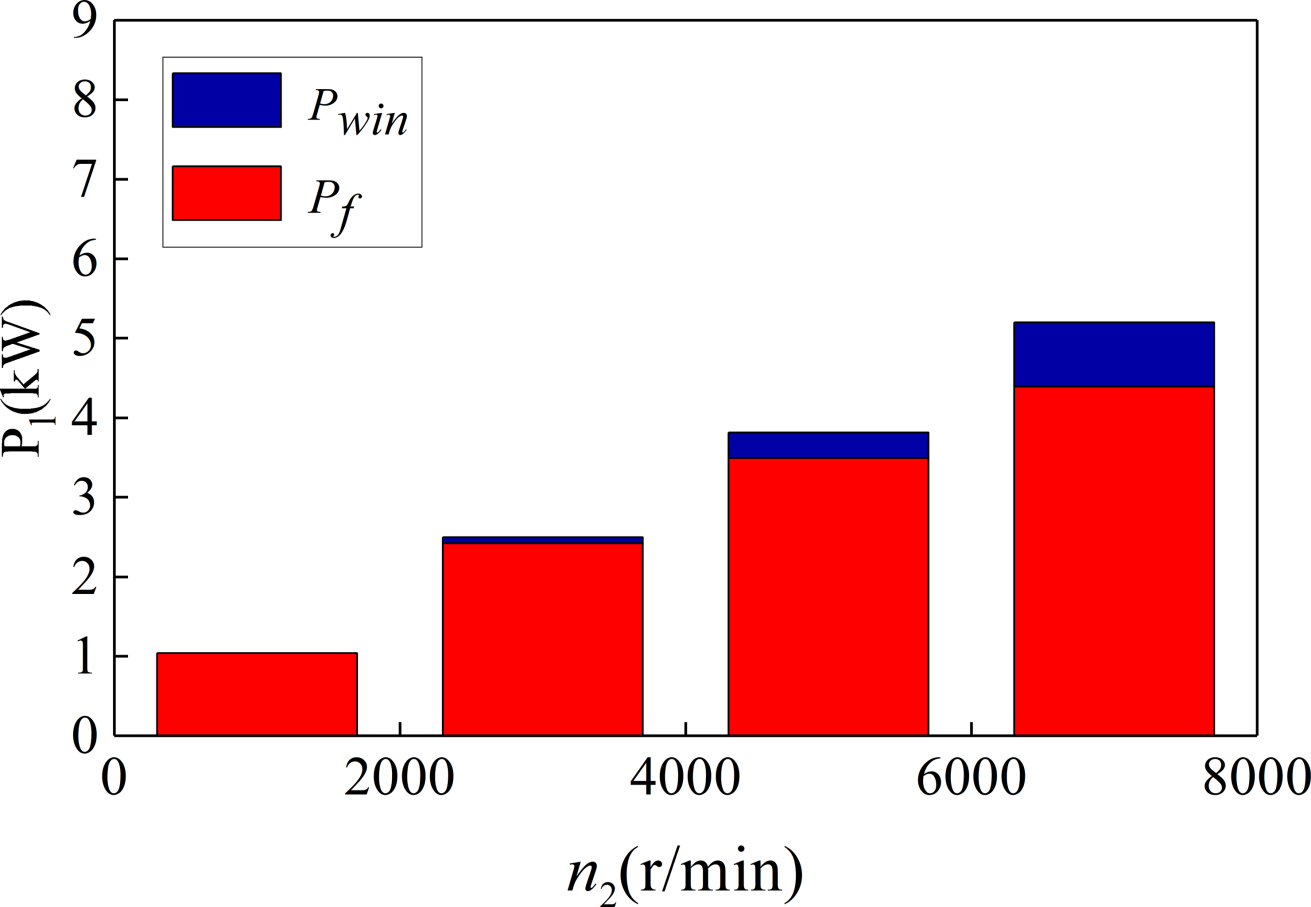
The power loss under the spray lubrication (*T*_2_ = 19950N·m).

**Fig** 16 and **Fig** 17 show the change of power loss with the change of pinion speed. **Fig** 16 is the oil-immersed lubrication, and **Fig** 17 is the spray lubrication.

It can be seen from **Fig** 16 and **Fig** 17 that the friction power loss (*P*_*f*_), wind resistance loss (*P*_*win*_), churning loss (*P*_*ch*_) of the spur-face gear are all increased with the increase of pinion speed. And the proportion of churning loss (*P*_*ch*_) and wind resistance loss (*P*_*win*_) in the total loss also increases with the increase of pinion speed. When the rotational speed is too high, churning loss (*P*_*ch*_) may exceed the friction power loss (*P*_*f*_). Therefore, spray lubrication is usually used when the speed exceeds 2000r/min-3000r/min.

**Fig 18:**
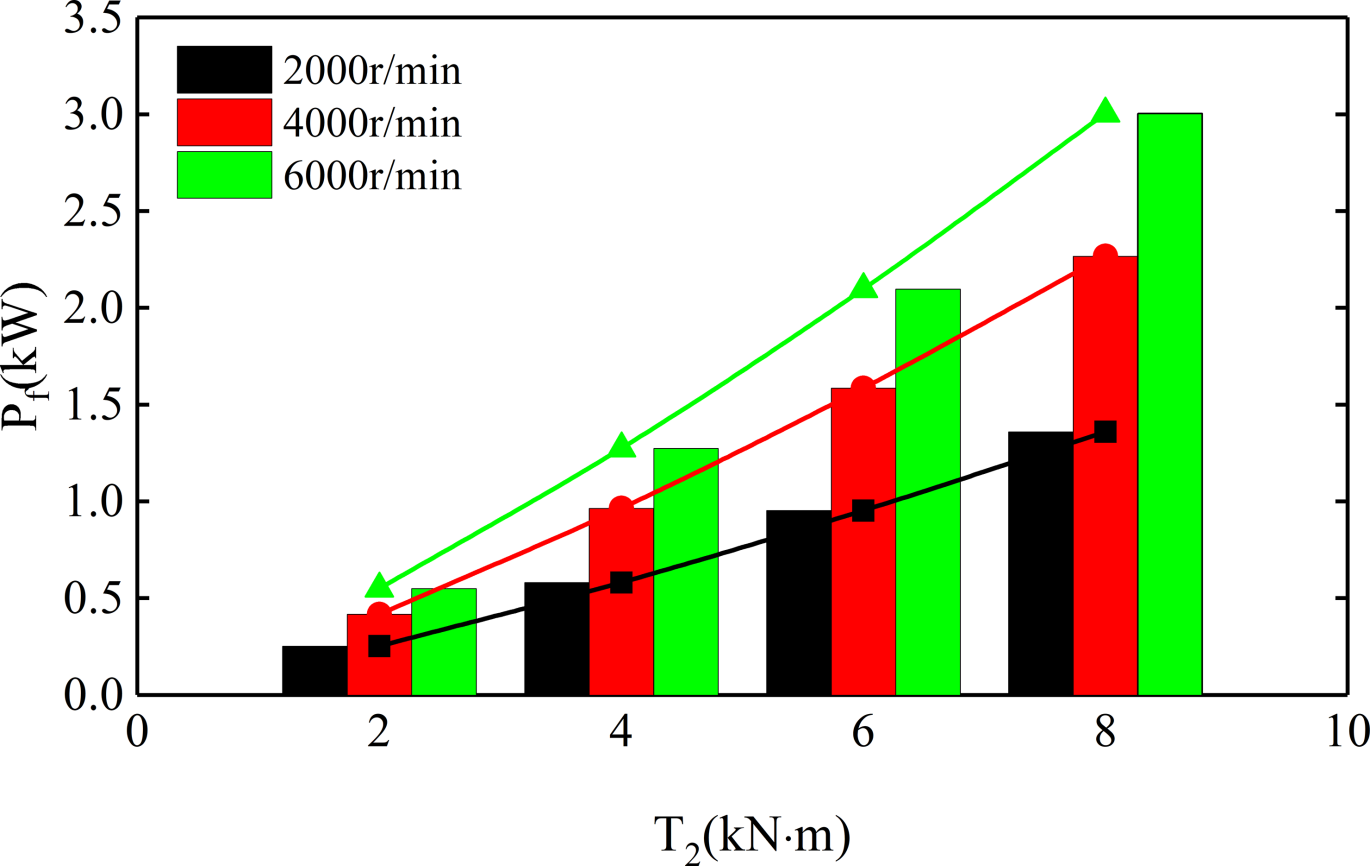
Sliding friction power loss.

**Fig 19:**
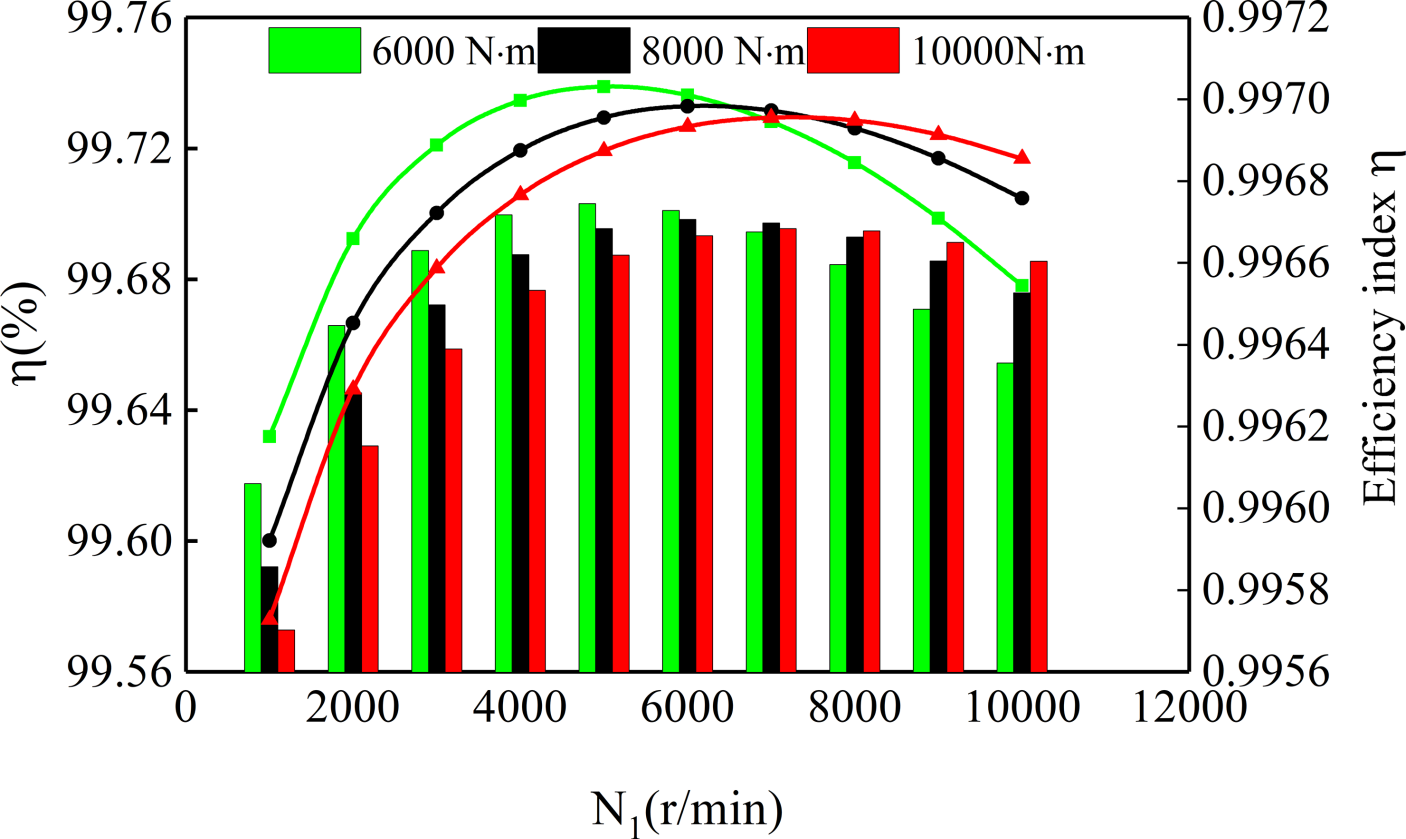
Gear meshing efficiency.

**Fig** 18 shows the variation of sliding friction power loss with torque of spur-face gear under different speeds. The power loss of sliding friction is related to rotation speed and torque, and increases with the increase of rotation speed and torque. When the torque changes from 2000 N·m to 8000 N·m, the sliding friction power loss increases from 500W to 3000 W. And the greater the speed, the greater the loss of power. **Fig** 19 shows the curve of gear transmission efficiency varying with spur-face gear speed under different torque. According to **Fig** 19, the efficiency increases first and then decreases with the increase of speed. When the speed is 0~7132r/min, the efficiency increases with the speed increase, and decreases with the increase of torque. When the speed is greater than 7132r/min, the efficiency decreases with the speed increase, and increases with the increase of torque.

## Verification of theoretical calculation model

In order to verify the correctness and feasibility of the mathematical model proposed above, the theoretical calculation model is compared with the experimental data of the power loss of the planetary transmission gear system of the Ohio State University, which is completed by Kahraman (Reference [9]). This experiment experimental proposed by Kahraman study investigates the contributions of the key components of load independent power losses of planetary gear sets.

The basic gear parameters given by the experiment are shown as shown in Table 3

**Table 3:** The basic design parameters of the test a gear set.

**A**			
**Parameter**	**Sun**	**Planet**	**Ring**
Number of teeth	73	26	125
Normal module [mm]	1.81	1.81	1.81
Pressure angle [deg.]	23	23	23
Helix angle [deg.]	13.1	13.1	13.1
**B**			
**Parameter**	**Sun and Planet**		**Planet and Ring**
Center distance [mm]	92.1		92.1
Active face width [mm]	25		25

The experimental each test of Reference[9] was conducted by starting up the test machine at the desired test speed and recording (i) input torque to the gearbox T, (ii) planet carrier speed *ω*_c_, and (iii) the temperatures at the fluid system reservoir and the inlet to the shaft lubrication system. All data was then averaged over the test period to arrive at a set of representative values of *T*, *ω*_*c*_, and reservoir and shaft inlet temperatures for each test. Each test was run for a period of 10 min. The total spin power loss of the gearbox within the tested configuration was then computed as *P* = *ω*_*c*_ *T*. Each test configuration was run at two different oil temperatures of 40°C and 90°C. Spin power loss planetary gear configuration test matrix in Table 2 in Reference [9].

**Fig 20:**
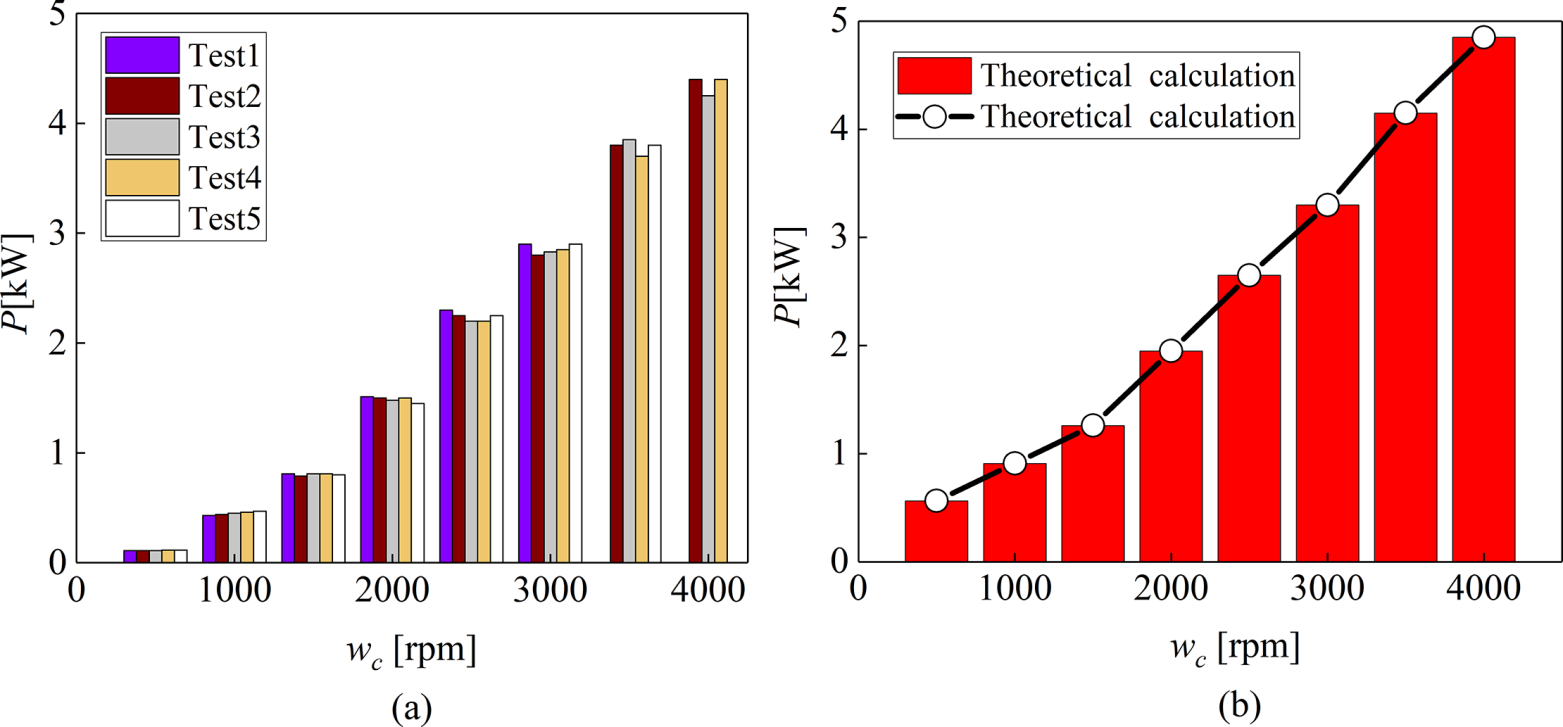
Comparison of Measured power losses and theoretical power loss calculation. (a) Measured power losses from Test 1A repeatability tests at 90°C. (b) Theoretical power loss calculation.

The experiment gave five sets of test data in accordance with the test 1A at 90°C, as shown in **Fig** 20(A). **Fig** 20(B) shows the power loss calculated by the mathematical model mentioned in this paper.

As can be seen from **Fig** 20, there is little difference between the theoretical calculation data and the experimental data, and the trend is the same. The value of power loss increases with the increase of rotational speed. The value of the theoretical data is larger than that of the experimental data, because the values calculated in theory are ideal parameters.

**Fig 21:**
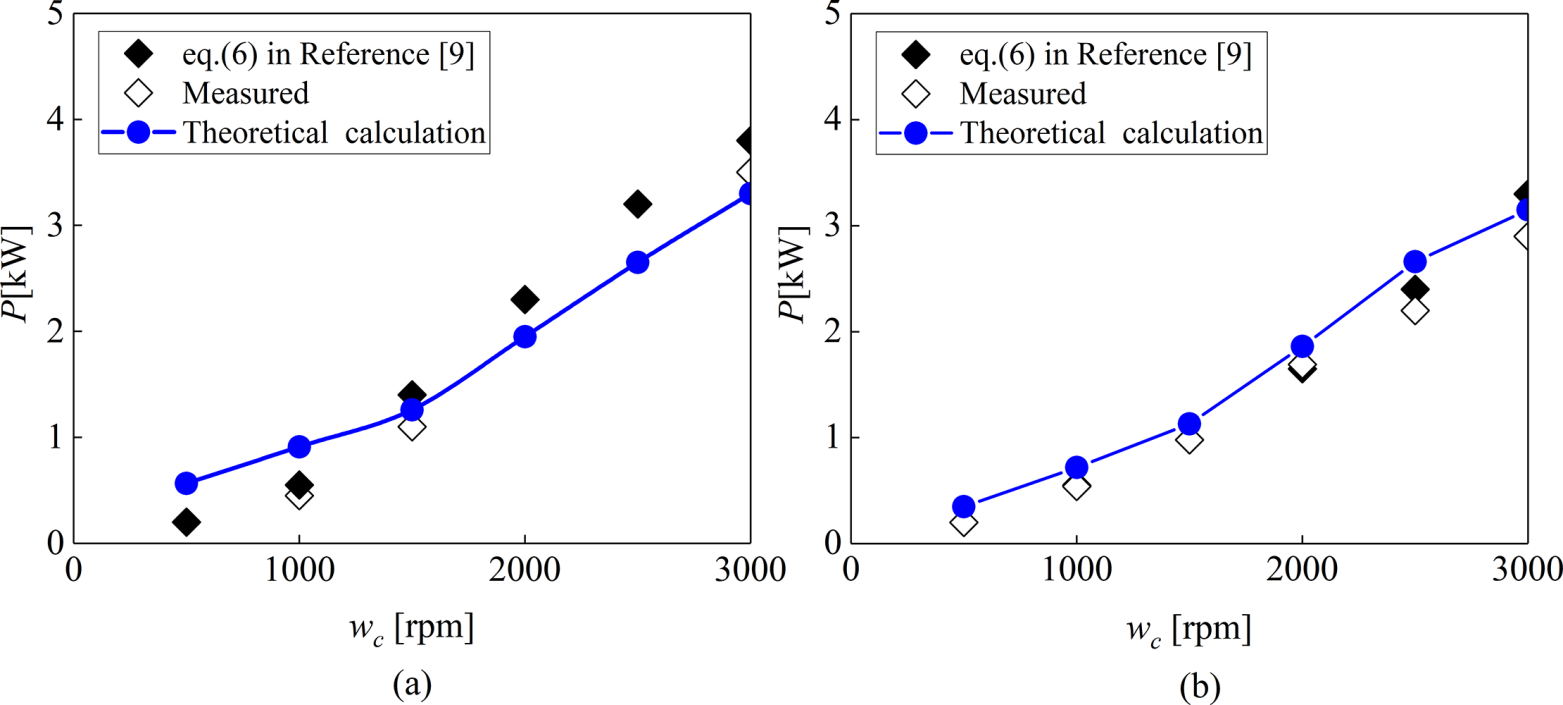
Comparison of Measured, Eq (6) in Referennce[9] and theoretical power loss calculation. (a) at 40°C. (b) at 90°C.

In the Reference [9], the total power loss value at 40° and 90° is recorded by experimental test, as shown in **Fig** 21. And in **Fig** 21, the data values of the theoretical calculation are given. In **Fig** 21, have a comparisons of the total power loss calculated from its components using Eq (6) to the actual measurements from test 1A at (a) 40°C and (b) 90°C. The Eq (6) in reference [9] is expressed as *P* = *P*_*ds*_+*P*_*dc*_+*N*(*P*_*ps*_+*P*_*pr*_)+*N*(P_*vb*_+*P*_*gb*_), The specific meaning is shown in reference [9].

Through **Fig** 21, it can be seen that the difference between the values of data measurement and the theoretical analysis is about 5%, and the data trend is consistent, which verifies the accuracy of the mathematical model proposed in this paper.

## Conclusions

Based on the theory of Elastohydrodynamic lubrication, this paper presents a method for calculating the Meshing Efficiency of Spur-Face gear. The development of thermal Elastohydrodynamic lubrication (TEHL) theory and its numerical solution makes the thermal Elastohydrodynamic lubrication (TEHL) theory be used to calculate the transmission efficiency of Spur-Face gear, and improve the calculation accuracy of gear transmission efficiency.

The conclusions are as follows:

1. The tooth contact analysis (TCA) and the loaded tooth contact analysis technique (LTCA) are used to simulate the meshing process of the straight tooth face gear, which can better show the mechanical characteristics of each meshing position on the tooth surface, and better describe the sliding friction coefficient at different positions on the tooth surface.
2. The coefficient of sliding friction at different positions on the tooth surface is not the same, and the sliding friction coefficient at the same position on the tooth surface is not the same under different working conditions. Therefore, calculating the gear meshing efficiency using the method of average friction coefficient may have calculation errors.
3. Torque and rotational speed are important factors affecting the friction coefficient of the sliding tooth surface. The coefficient of sliding friction of the tooth surface increases with the increase of the torque, and decreases with the increase of the rotational speed.
4. Furthermore, the sliding friction coefficient of the tooth surface is an important factor affecting the gear meshing efficiency. The effect of torque and speed on the meshing efficiency is achieved by affecting the sliding friction coefficient of the tooth surface. Considering only the sliding friction power loss, the gear meshing efficiency increases with the reduction of the sliding friction coefficient.

The theoretical data and the experimental data of reference [9] are compared, which verifies the accuracy of the mathematical model proposed in this paper.
